# Fungal Identifier (FId): An Updated Polymerase Chain Reaction–Restriction Fragment Length Polymorphism Approach to Ease Ascomycetous Yeast Isolates’ Identification in Ecological Studies

**DOI:** 10.3390/jof10090595

**Published:** 2024-08-23

**Authors:** Silvia Abbà, Beatrice Valentini, Irene Stefanini

**Affiliations:** Department of Life Sciences and Systems Biology, University of Turin, 10123 Turin, Italy; silvia.abba@unito.it (S.A.); beatrice.valentini@unito.it (B.V.)

**Keywords:** fungi, molecular, PCR-RFLP, identification, microbial ecology

## Abstract

Culturomics has been temporarily exceeded by the advent of omics approaches such as metabarcoding and metagenomics. However, despite improving our knowledge of microbial population composition, both metabarcoding and metagenomics are not suitable for investigating and experimental testing inferences about microbial ecological roles and evolution. This leads to a recent revival of culturomics approaches, which should be supported by improvements in the available tools for high-throughput microbial identification. This study aimed to update the classical PCR-RFLP approach in light of the currently available knowledge on yeast genomics. We generated and analyzed a database including more than 1400 ascomycetous yeast species, each characterized by PCR-RFLP profiles obtained with 143 different endonucleases. The results allowed for the in silico evaluation of the performance of the tested endonucleases in the yeast species’ identification and the generation of FId (Fungal Identifier), an online freely accessible tool for the identification of yeast species according to experimentally obtained PCR-RFLP profiles.

## 1. Introduction

The advent of next-generation approaches has greatly impacted the microbiology field by providing relatively accessible tools suitable for the description of microbial populations in multiple environments [1,2,3]. These new potentialities exceeded the culturomics approaches, which were initially neglected mostly because of the demanding expertise and time requests and the impossibility of identifying non-cultivable microorganisms. The early developed approach, metabarcoding, based on the sequencing of marker genes suitable for the identification of microbial species (e.g., the 16S rRNA gene for prokaryote identification), provided a successful tool to detect microbial species previously undetected through culturomics [1]. Metabarcoding earned great success, opening up new perspectives for non-specialized researchers thanks to the availability of well-defined and easily reproducible experimental protocols and bioinformatics tools for the analysis. In microbiology ecology studies, metabarcoding has provided a successful way to estimate the microbial alpha and beta diversities and general differences between communities. Metagenomics analysis, exploring the entire DNA set present in a given sample, further expanded the metabarcoding potentials by allowing for the investigation of the microbial functional genome. Nevertheless, these powerful approaches still suffer from some limits when studying natural systems with high diversity, often presenting a relevant environmental noise (e.g., DNA from soil or other matrixes) and including a wide range of physiological states of living organisms. In addition, the increased amount of data and the improved knowledge of microbial populations arising from the application of these new approaches evidenced that the identification of microbial species based on the sequence of a single gene was not always possible nor reliable due to the presence, in some organisms, of multiple dissimilar copies of the gene markers [4]. This aspect was even more severe in the case of fungal community investigations, considering that an agreement was reached only recently on the use of the ITS1-5.8S-ITS2 region as a marker for species identification [5]. Metabarcoding was widely adopted for the description of microbial populations, but the necessity of delving into the information, calling for detailed studies of the implication of different compositional profiles in the studied settings, rapidly became clear. This leads to the development of tools aimed at predicting the potential functions bore by a given microbial population assuming phenotypic uniformity within the taxonomic level [3,6]. Yet, dramatic phenotypic differences were appreciated among individuals belonging to the same taxonomic level (up to the species level), hence calling at the same time the urgency of higher sequencing resolution compared to the single-marker one [4] and the need to preserve the culturomics approach [7]. Metagenomics approaches, based on the sequencing of the entire set of DNA available in the sample, partially improved this deficiency by allowing for the identification of sequences coding for proteins functionally described. Alas, the approach requires great cost and technical investments, making them not easily accessible, and still does not ensure the actual occurrence of the identified function in the studied settings (i.e., gene expression is regulated by multiple events and factors). Furthermore, the major flaw of NGS or, in general, molecular-based approaches stands in the impossibility of obtaining the actual isolate, which can be further investigated for phenotypic and metabolic features that can help in further understanding microbial species evolution, variation, and selection in the studied environment. Overall, these drawbacks and limitations of metabarcoding and metagenomics approaches in assessing and experimental testing inferences about ecological roles and evolution of microorganisms, especially in light of the studied system, lead to a revival of culturomics approaches [7]. When adopting culturomics for exploring microbial populations from an ecological perspective, the most relevant approach is obtaining a characterization of the entire population, hence resulting in the simultaneous isolation of the broadest range of microorganisms not selected by the isolation approach. The taxonomic identification of this large set of isolates is an economical- and time-demanding step. Phenotype microarrays, such as Biolog [8], have been proposed to simultaneously identify microbial isolates and characterize their phenotypic (mostly metabolic). Despite representing a useful resource, this technique is still only partially scalable to high-throughput levels (hence requiring an initial screening of the isolates) and is currently applicable to a restricted group of more characterized microorganisms (mostly pathogenic prokaryotes). More recently, MALDI-TOF MS (matrix-assisted laser desorption/ionization–time of flight mass spectrometry) approaches for the identification of microorganisms by comparing the peptide or protein profiles to reference strains [9], recently implemented with imaging data (mass spectrometry imaging, MSI) [10], have been developed. While providing information suitable for precisely identifying the microorganism, these approaches require specific expertise and a substantial initial investment (purchasing the instrument) [10]. Easily accessible and rapid services for Sanger sequencing of the genomic markers could support this process, but a first step of high-throughput screening to assess the presence of multiple isolates belonging to the same species (potentially clones of the same individual) is fundamental to speed up and ease the investigation. Aiming at this, fingerprinting techniques such as microsatellite-primed PCR (MS-PCR) [11] and Polymerase Chain Reaction–Restriction Fragment Length Polymorphism (PCR-RFLP) [12] have been optimized to gather information on the taxonomy of large numbers of yeast isolates. MS-PCR fingerprinting, based on the amplification of microsatellites, was shown to lead to potential misidentification due to the high similarity of profiles among multiple species [11], but it can be applied when studying yeast populations of predictable composition [13]. In the field of yeast ecology, the PCR-RFLP approach proposed by Esteve-Zarzoso and colleagues in 1999 [11] was greatly appreciated and adopted by most yeast-investigating researchers. The interest in fungal populations in multiple environments ranging from soil [14], biomasses [15], insects [16,17], marine water [18], to crops [19], often resulting in the identification of yeast isolates with relevant phenotypic characteristics suitable for biotechnological applications, dramatically demanded an update of the PCR-RFLP approach. When analyzing samples rich in fungal species (e.g., early-stage wine must sample), thousands of yeast colonies can be isolated, and the target species are in most cases not phenotypically or metabolically discernible from other species. The PCR-RFLP approach can reduce the number of isolates to be further processed and investigated using additional tests. So far, the yeast species identifiable through PCR-RFLP were limited to a small range of species commonly found in specific environments (mostly fermentation) [11,12]. The identification of new yeast species, leading to the re-evaluation of the previously predicted extent of fungal species diversity [20], and the exponential increase in genomic sequences (currently 14,199 Ascomycota genome sequences available on NCBI, of which 11,220 were released over the last five years), highlighted the need to evaluate the applicability of the PCR-RFLP on this expanded range of species.

With this study, we aimed to provide an objective evaluation and update of the PCR-RFLP approach in light of the currently available knowledge on yeast genomics. We generated and analyzed a database including more than 1400 yeast species, each characterized by PCR-RFLP profiles obtained with 143 different endonucleases. The comparative analysis of the PCR-RFLP profiles allowed for the in silico evaluation of the performance of the tested endonucleases in the yeast species’ identification. Furthermore, we also designed a free online accessible tool for the identification of yeast species according to PCR-RFLP profiles. This new tool, different from already available tools (e.g., Yeast-ID.org) [21], provides profiles for a greatly larger number of yeast species, allows for the combination of multiple endonuclease profiles, and the identification of the best endonuclease suitable for the identification of the entire set of investigated yeast species, hence facilitating the identification of the target species in large sets of yeast isolates.

## 2. Materials and Methods

### 2.1. Ascomycete Genomes and Sequence Retrieval

To retrieve the sequences of the ITS1-5.8S-ITS2 genomic region, a total of 2351 genomes of Ascomycete fungi were downloaded from the NCBI’s genome database [22], using the filter for reference genomes (April 2023, Table S1). In the case of multiple genomic sequences for the same species, the most complete one (according to the level of assembly—contigs, scaffold, chromosome) was selected. After the download, the ITS1-5.8S-ITS2 was searched in the genome sequences through a local blastn analysis, using the *Saccharomyces cerevisiae* ITS1-5.8S-ITS2 sequence as a query and default parameters for the search [23]. The blastn results were inspected to search for positive and high-confidence matches (identity > 90%, E-value closest to 0). Then, the contig/scaffold/chromosome which had shown a match with the reference ITS1-5.8S-ITS2 sequence was extracted from the full genome fasta file for each genome sequence by using awk. The selected contig/scaffold/chromosome was then inspected for the extraction of the precise ITS1-5.8S-ITS2 region that could be amplified through PCR with the primers ITS1 (5′-TCCGTAGGTGAACCTGCGG-3′) and ITS4 (5′-TCCTCCGCTTATTGATATGC-3′) [12]. Aiming at this, cutadapt was used [24] with the following parameters: -n 2 --overlap 18 (the minimum length of the overlap between the genomic sequence and the primers sequence) -e 0.1 (the number of errors in the match divided by the length of the matching part of the primers). The resulting sequences were individually inspected and, if too short (<200 bp) or no sequences were found, the analysis was repeated on the complement reverse sequences, eventually relaxing the parameter concerning the maximum number of allowed errors/mismatches (-e 0.2). If the primer sequences were found in the reverse complement genomic sequences, the sequences resulting from the cutadapt analysis were converted into their reverse complement. 

### 2.2. In Silico Endonuclease Restriction

The list of endonucleases was retrieved from the REBASE database [25], and the corresponding restriction sites were manually annotated. Custom Python scripts were generated to quantify the length of the ITS1-5.8S-ITS2 regions (amplicons, definition_of_amplicon_size.py, File S1), perform in silico the activity of the endonucleases, and obtain the sequence and length of the fragments resulting from the digestion of the previously obtained ITS1-5.8S-ITS2 regions (get_length_of_fragments.py, File S1). In the case of restriction sites with ambiguous nucleotides, all the alternative sequences were searched. The resulting restriction profiles, obtained with the Python script described in Section 2.2 and encompassing, for each species, the lengths of the fragments obtained with the in silico endonuclease restriction, were imported and analyzed in R [26]. For each endonuclease, identical profiles were considered indistinguishable species.

### 2.3. Valuation of the Experimental Error and Calibration of the Model

To make the information obtained in silico suitable for comparison with experimental data, the experimental error arising from the quantification of the fragment lengths was evaluated. For calibration purposes, any DNA molecule of known size could be used. We used amplicons and fragments obtained from PCR-RFLP on ITS1-5.8S-ITS2 both to make sure to capture the molecule length ranges of the procedure and to provide an experimental protocol that will be suitable for using the tool developed in this study. PCR for amplification of the isolates’ ITS1-5.8S-ITS2 region was carried out as previously described [27]. Briefly, the ITS1 (FW, 5′-GTTTCCGTAGGTGAACTTGC-3′) and ITS4 (RV, 5′-TCCTCCGCTTATTGATATGC-3′) primers were used. The GoTaq DNA polymerase (PROMEGA) was used with the following thermal cycle: 95 °C for 1 min, (95 °C for 30 s, 53.6 °C for 30 s, and 72 °C for 1 min and 30 s) repeated for 35 cycles, 72 °C for 10 min. The PCR products, upon assessment of the size on a 1% agarose gel, were digested with the HaeIII endonuclease for 1h at 37 °C. The digested PCR products were run on 2.5% agarose gel stained with EuroSafe fluorescent nucleic acid stain (Euroclone) to assess the size of the fragments. Images of the gel were analyzed with the GeneAnalyzer 19.1 software (Istvan L.Jr. and Istvan L.Sr., www.gelanalyzer.com accessed on 1 January 2023) with the following settings: automatic detection of lines, automatic detection of peaks on all lanes (peak threshold = 2, peak min height = 2, peak max-width in % of lane profile length = 20). Two DNA ladders (Quick-Load^®^ Purple 100 bp DNA Ladder (NEB) and 100 bp DNA Ladder GL (genespin)) and profiles from different yeast species (complete list in Table S4) were evaluated. Yeast species were confirmed through Sanger sequencing of the ITS1-5.8S-ITS2 region (the NCBI accession IDs are shown in Table S4). The resulting data were used to develop a mathematical model to predict the experimental error of the fragment or amplicon length measurements (in other words, to determine the permissive range of sizes corresponding to the measured amplicon/fragment length). Aiming at this, two approaches were adopted: linear regression with least squares errors, hereinafter referred to as “lm”, and linear regression with correction for correlation in the error terms (corAR1) and correction for within-group heteroscedasticity (VarPow), hereinafter referred to as “VarPow”. 

### 2.4. Testing the Model with Experimental Data

The models obtained as described in Section 2.3 were validated with experimental data. Aimed at this, experimental data obtained for this study by applying the protocol described earlier and from previously published studies [12,21,28,29,30,31,32] were used. In total, 534 experimental amplicons or PCR-RFLP profile data were used for this validation. The experimental data were fed to the models and two indexes were used to evaluate the models’ performance: (i) identification, successful if the yeast species associated with the experimental data were found in the list of species selected by the model; precision, defined as the number of additional species included in the list of species selected by the model (the higher the number, the lower the precision).

### 2.5. Identification of Yeast Species with Experimental Error

To further assess the performance of both lm and VarPow methods and to test the potential of all the available endonucleases, 300 yeast species were randomly selected, and the relative ITS1-5.8S-ITS2 amplicon length and restriction fragment profiles calculated in silico were used as the input for the analysis. For each query species, the process of identification consisted of the following: selecting the species with amplicon lengths within the confidential error range according to the selected approach; selecting, among the species selected at the first stage, the species showing restriction profiles with the highest match with the profile of the query species (by evaluating whether the fragments, corrected according to the selected error model, corresponded to the fragments in the restriction fragments reference database); and selecting, among the species selected at the second stage (selection on the first restriction profile), the species showing restriction profiles with the highest match with the query profile of the query species. The approach was performed for all the possible combinations of endonucleases, also considering the selection order (e.g., first selection with enzyme 1 and second selection with enzyme 2, but also first selection with enzyme 2 and second selection with enzyme 1). The following combinations of analyses were performed: VarPow on amplicon length and lm on restriction profiles; VarPow on both amplicon length and restriction profiles. Several indexes were evaluated: (i) % of identification, (ii) Median Improvement, (iii) Median n_secSel, (iv) Precise Ident, (v) Ranking Identification, (vi) ranking_FD, (vii) Quartile Identification, (viii) quartile_FD, (ix) Overall Ranking. Details of the indexes are described in Table S7 reporting the analysis results.

### 2.6. Testing the Combination of Multiple Profiles

According to the results of the model test on one or two restriction profiles, a selection of enzymes was further tested to improve the precision of the identification by combining 3 to 5 profiles. The selection of enzymes was imposed by computational limitations, as testing all the possible combinations of five profiles would result in more than 55 billion tests (143*142*141*140*139) for each yeast species evaluated. The enzymes were selected as those that, when used alone, allowed for the identification of the correct yeast species and resulted in less than 50 species selected together with the correct one (resulting in 80 enzymes selected). Considering the results of the tests with two enzymes, the enzymes were further selected as those whose profile, when used in combination with another profile, resulted in the precise identification of the largest number of yeast species. These two selections resulted in 80 enzymes being used for the first selection based on profiles and 22 enzymes (also including some of the 80 enzymes selected for the first level) being used for the selections with additional profiles. All the possible couples, triplets, quartets, and quintuplets were tested on 100 randomly selected yeast species, proceeding as described for the analysis of enzyme couples (Section 2.5). The results were evaluated according to the percentage of yeast species correctly identified and the number of additional species selected by the process. The frequency of each enzyme among the combinations resulting in the precise identification of the correct yeast species was evaluated, and the enzymes more frequently included were considered to be the best-performing ones. The combinations including these enzymes were further investigated to identify the best combinations according to the same indexes.

## 3. Results

### 3.1. Sequence Database

**Figure 1:**
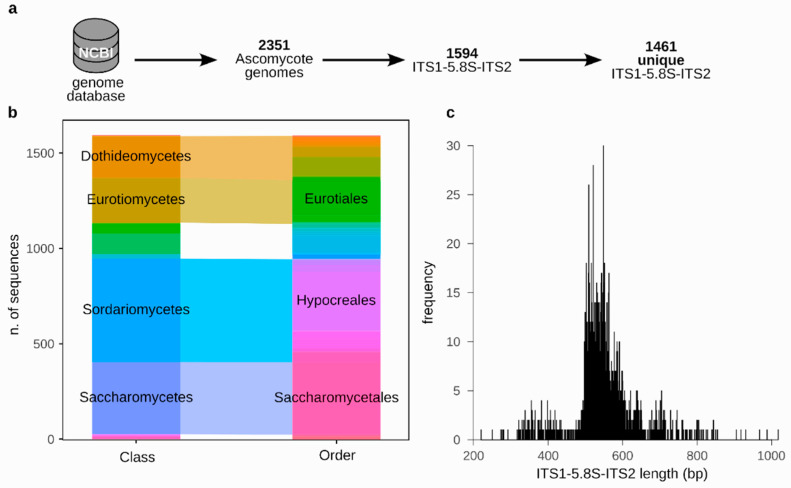
Characteristics of the database. (**a**) Process of sequence selection. (**b**) Composition of the sequence database in terms of classes and orders. (**c**) Distribution of the length of the ITS1-5.8S-ITS2 region, calculated in silico, among the sequences composing the database.

At the time of the analysis, the NCBI database included 2351 reference genome sequences from different Ascomycete species (Figure 1a). Complete ITS1-5.8S-ITS2 sequences were found, using a blastn search against the *Saccharomyces cerevisiae* sequence, in only 1594 out of the 2351 available Ascomycete genome sequences. Of the retrieved sequences, 206 were identical among different species, resulting in 1461 different sequences (Table S1). The dataset included species belonging to the Sordariomycetes class (542 sequences, 37.10% of the dataset), followed by Saccharomycetes (378 sequences), Eurotiomycetes (234 sequences), Dothideomycetes (219 sequences), and Leotiomycetes (106 sequences) (Figure 1b, Table S1). The most represented orders were Saccharomycetales (378 sequences), Hypocreales (309 sequences), and Eurotiales (176 sequences) (Figure 1b, Table S1). The dataset included a total of 179 families, with the most abundant being Nectriaceae (185 sequences), and 465 genera, with the most abundant being *Fusarium* (145 sequences) (Table S1).

We assessed the differences among the length of the ITS1-5.8S-ITS2 region of the genomes composing the database. The 1461 unique sequences showed 359 different lengths (median value = 544), with the shorter sequence being the *Pichia nakasei* species (220 bp) and the longest sequence being the one of *Amauroascus niger* (1018 bp, Table S2, Figure 1c). Hence, as previously observed [12], just considering the length of the ITS1-5.8S-ITS2 is not a characteristic allowing for the identification of the yeast species.

### 3.2. Restriction Fragments Profiles Obtained with Endonucleases In Silico

**Figure 2:**
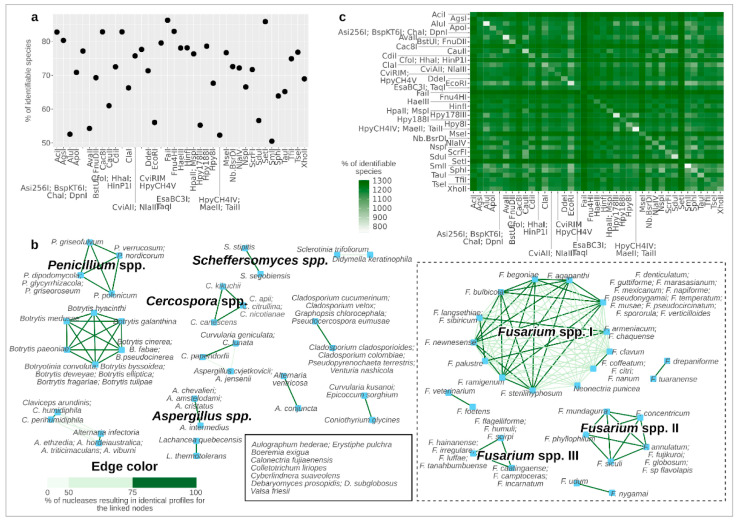
Database information. (**a**) Comparison of identifiable yeast species according to the PCR-RFLP obtained through the best-performing endonucleases. To improve the readability of the image, the plot shows only the endonucleases, resulting in more than 50% of yeast species being identifiable; the complete plot is in Figure S2. (**b**) Species showing indistinguishable profiles utilizing PCR-RFLP analysis. Each network shows the groups of yeast species showing identical profiles according to PCR-RFLP based on the tested endonuclease. The edge color indicates the percentage of tested enzymes that resulted in an identical profile shared by the two connected yeast species (nodes). The dotted rectangle includes groups of indistinguishable species belonging to the *Fusarium* genus. (**c**) Percentage of species identifiable according to the combination of PCR-RFLP profiles of two endonucleases. The color of the heatmap cells indicates the number of yeast species identifiable thanks to the combination of the profiles obtained with the endonucleases indicated in the corresponding row and column. To improve the readability of the image, the plot shows only the endonucleases, resulting in more than 50% of yeast species being identifiable; the complete plot is in Figure S3.

To proceed with the identification of the best PCR-RFLP approach to identify the highest number of fungal species, we explored the molecular profiles obtained using in silico restriction of the ITS1-5.8S-ITS2 region by 147 endonucleases recognizing different restriction sites (Table S3). The restriction sites of four tested endonucleases were not present in at least one of the ITS1-5.8S-ITS2 sequences (I-CeuI, I-SceI, PI-PspI, and PI-SceI, Table S3) and were then excluded from the analysis. Aiming at identifying the optimal endonuclease for the PCR-RFLP analysis, the efficacy of each enzyme was evaluated as the number of different restriction profiles, calculated as the fragments of DNA obtained through the endonuclease activity (hence considering the size and number of fragments). The number of species unequivocally identifiable thanks to the obtained PCR-RFLP profile was also evaluated for each tested endonuclease as the number of profiles associated with a single species. As expected, the percentage of observed profiles exceeded the number of identifiable species for every endonuclease (Figure S1). The best-performing enzyme, resulting in 1336 different profiles and identifiable 1260 species (86.24% of the dataset), was FaiI, followed by SetI (identifiable 1254 species, 85.83%) and Fnu4HI (1212 identifiable species, 82.96%) (Table S3). CfoI, HaeIII, and HinfI, the three enzymes previously proposed for PCR-RFLP analysis [12], despite not performing as well as other enzymes, were still capable of identifying a great portion of yeast species, with 82.82% (1210 identifiable species), 78.03% (1140 identifiable species), and 78.10% (1141 identifiable species) of species being, respectively, identified (Figure 2a, Figure S2). A total of 68 out of the 1461 analyzed yeast species could not be identified through the profiles obtained with any tested endonucleases, as the respective restriction profiles were identical to at least one other species. Most of these indistinguishable species showed profiles identical to species belonging to the same genus, with several groups of identical profiles characterizing the *Fusarium* genus (in the box limited by the dotted line in Figure 2b). Two groups of species employing network analysis showed the highest number of components: the one composed of *Fusarium* spp. (identified as “*Fusarium* spp. I” in Figure 2b) and a network including *Botrytis* and *Botryotinia* species. Among these indistinguishable species were found to share their profiles with different species depending on the used endonuclease, hence suggesting that their identification could be resolved by using combinations of PCR-RFLP obtained with multiple endonucleases (nodes connected with light green edges in Figure 2b).

To improve the possibility of identifying the largest number of yeast species, we assessed the number of species unequivocally identifiable thanks to the combination of PCR-RFLP profiles obtained separately with two different endonucleases. The best combination of profiles, obtained through FaiI and SetI endonucleases, resulted in the identification of 1305 species (89.32%), hence improving the potential of both enzymes, which individually allowed for the identification of 1260 and 1254 species (Figure 2c and Table S4). The same analysis was carried out by combining the PCR-RFLP profiles obtained with three different endonucleases, and the results confirmed FaiI and SetI as the best-performing enzymes, but the addition of a third profile obtained with a different enzyme did not increase the number of identified species (Table S5).

### 3.3. Analysis of Experimental Data

The procedure described so far allows for the generation of a reference database including the PCR-RFLP profiles for all the available genomic sequences of yeast species. The profiles were obtained in silico, but experimental procedures cannot reach the precision required to detect the very small fragment length differences computationally observed among PCR-RFLP profiles. Hence, to expand the possibility of using FId to discriminate yeast isolates by using classical laboratory instrumentation (e.g., horizontal agarose gel electrophoresis), we first assessed experimentally the instrumental errors in the quantification of the length of PCR-RFLP fragments and then repeated the assessment of the protocol, as previously carried out with the in silico data.

First of all, we evaluated the experimental error in the definition of the length of DNA amplicons and fragments obtained in this study, as described in the Materials and Methods and previous studies [12,21,28,29,30,31,32], resulting in a dataset composed of 1183 measured and expected information on DNA molecule length (Table S5). By comparing the measured length with the expected length of each DNA molecule included in the dataset, we could observe that fragments shorter than 100 bp could not be observed or properly quantified and should hence be excluded from the analysis (Figure S4a). To buffer experimental errors in the definition of amplicon and fragment sizes, we modeled calibration curves and relative errors with two approaches, lm and VarPow (further described in the Materials and Methods section) (Figure S4b). Upon calibration of the two models, their performance was tested on experimental data obtained previously with the HaeIII endonuclease [27], and restriction profiles obtained with multiple endonucleases in previous studies [12,21,28,29,30,31,32], for a total of 534 different profiles (Table S6). Thanks to this analysis, we observed that whereas the VarPow approach guaranteed a higher capacity of identification (91.83% on amplicons, 81.05% on restriction patterns), similar to the one observed with in silico data for the analyzed enzyme (82.82%), and low precision (between 1 and 203 species identified together with the expected one, median = 14.5 species), the lm approach was less successful (7.84% on amplicons, 1.63% on restriction profiles), but more precise in identification (between 1 and 6 species, median 1 species) (Figure S4c). 

We then assessed the performance of the models in the identification of yeast species with all the endonucleases included in the dataset by randomly sampling 300 species and quantifying the number of species correctly identified by the models. Considering that the test on experimental data highlighted the greater identification success and lower precision of the VarPow model compared to the lm model, the VarPow model was used to select species according to the ITS1-5.8S-ITS2 amplicon size (to ensure the identification of the species). To contain the low precision observed for the VarPow approach, the identification based on restriction profiles was performed by testing both the VarPow and lm approaches (see Materials and Methods for further details). The results confirmed that VarPow was the best-performing approach, with 81 enzymes allowing for the identification of all the tested yeast species (Figure S5a). Three endonucleases (FaiI, SetI, and NlaIV) could not allow for the identification of the largest number of yeast species. To note, two out of these enzymes (FaiI and SetI) were identified as the best-performing endonucleases in the in silico approach, and the lack of identification including the experimental error can be ascribed to the fact that their activity resulted in profiles characterized by several fragments of length lower than 100 bp (61% and 81% of the entire set of fragments for FaiI and SetI, respectively), not properly detected experimentally and hence excluded from the analysis (Figure S6b). As previously observed, despite the high identification success of the VarPow approach, this method was also associated with low precision, as indicated by a large number of false positives (species identified as potentially matching the query information, Figure S5b). To improve the precision of the identification, we assessed whether, by combining PCR-RFLP profiles achieved with two different endonucleases, the identification through experimental data would improve. Aiming at this, we used a pipeline with a progressive selection of potential species according to the ITS1-5.8S-ITS2 PCR amplicon length, the PCR-RFLP profile obtained with a first endonuclease, and an additional PCR-RFLP profile obtained with a second endonuclease. The procedure was carried out with the VarPow model on profiles obtained in silico for 100 yeast species randomly picked from the database and processed with all the possible combinations of endonucleases (Figure S7, Table S7). Despite resulting in some enzyme couples selecting a large number of false positive yeast species (up to a median of 647), the VarPow approach allowed for the identification of every tested query species in 10599 combinations of endonucleases (52.20% of the total possible combinations) (Table S7). Using the endonuclease SetI as the first enzyme for selection resulted in the least successful combination, identifying between 23% and 25% of query species (Table S7). 

**Figure 3:**
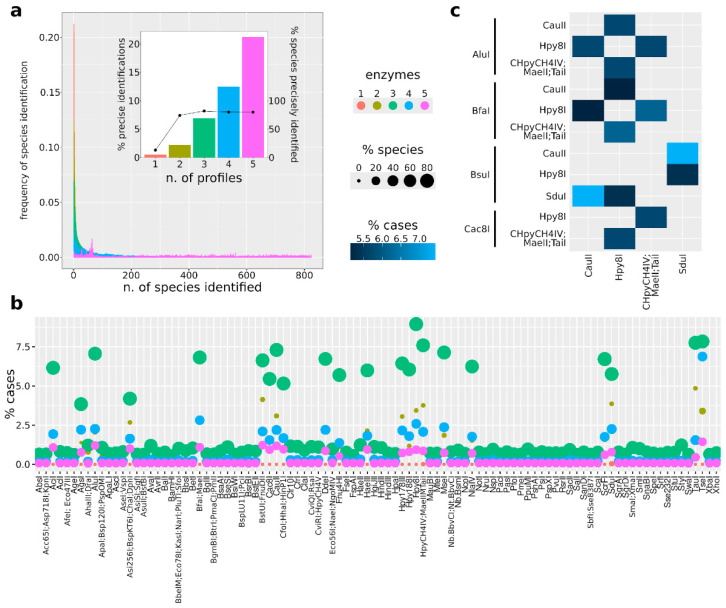
Results of multiple profile combinations. (**a**) Frequency of yeast species identified with single or multiple PCR-RFLP profiles. In the embedded plot, bars indicate the percentage of precise yeast species identifications (left y-axis) and the percentage of yeast species precisely identified with the combination of profiles (right y-axis). (**b**) Contribution of different endonucleases in precisely identifying the correct species. The percentage of cases indicates the percentage of the combination of profiles including the indicated enzyme. (**c**) Performance of the best triads of enzymes. The best-performing triads are shown, with the values indicating the percentage of triads among the best-performing ones including the enzymes indicated in the row and column labels.

To further improve the precise identification of yeast species, we performed the same analysis considering the combination of results from three, four, or five different endonucleases. The analysis was performed only considering the endonucleases that, as couples, resulted in 100% successful identification with less than 50 potential species identified and whose combination improved the precision by at least 25% compared to when used individually (Table S7). The overall percentage of combinations resulting in the precise identification of yeast species greatly improved with the increase in the number of profiles used for the analysis, with 20% of five profile/enzyme combinations resulting in the precise identification of the correct species (Figure 3a). However, all the yeast species precisely identifiable were uniquely identified already with the combination of three profiles (Figure 3a), hence indicating that the combination of three profiles is sufficient. The profiles obtained by twenty-two endonucleases were most frequently present in the triads, resulting in precise identifications (Figure 3b). By further delving into the results obtained with the most frequent endonucleases, the best-performing triads (among the highest fraction of the triads) included AluI, BfaI, BsuI, Cac8I, CauII, Hpy8I, HpyCH4VI/MaeII/TaiI, and SduI (Figure 3c). 

Finally, we have tested our approach by using PCR-RFLP profiles obtained in previous studies [12,21,27,29,30,32,33,34] and evaluating the capability of identifying the correct yeast species and the number of false positives. Out of the 90 samples analyzed (described through PCR-RFLP profiles obtained with various endonucleases, Table S8), 81 were correctly identified, with a median of four false positives per identification. To note, the nine cases of missing identification were associated with species whose profiles did not match with previously published profiles (e.g., *Candida glabrata*, *Candida mesenterica*, *Saccharomyces cerevisiae*, *Torulaspora delbrueckii*). Furthermore, the BLAST analysis of the ITS1-5.8S-ITS2 yeast species not precisely identified with our approach (with false positive identification) revealed several cases of inaccurate identification through the genomic region’s sequencing (Table S8). Our approach was also tested to confirm the capability of identifying species when considering potential intra-specific mutations in the ITS1-5.8S-ITS2 region. Aiming at this, we gathered ITS1-5.8S-ITS2 sequences for highly represented species (*Aspergillus fumigatus*, *Aspergillus niger*, *Candida albicans*, and *Saccharomyces cerevisiae strains*; 500 sequences for each species) and confirmed the capability of the approach in correctly identifying the species even in the case of sequence gaps (possibly originating from sequencing errors, Figure S8). Concerning *A. fumigatus* and *A. niger*, the ITS1-5.8S-ITS2 sequence was highly conserved, hence not influencing the activity of endonucleases. For *C. albicans*, a few SNPs were found among the randomly selected sequences, but these SNPs did not change the length of the ITS1-5.8S-ITS2 sequence nor the length of potential endonuclease restriction fragments (as the SNPs were not found in restriction sites), nor did they introduce non-traceable changes (buffered by the VarPow correction) (Figure S8). The comparison of *S. cerevisiae* strains’ sequences highlighted the presence of variations in restriction sites (StuI, MfeI, BseSI, ApaI/Bsp120I/PspOMI) in regions presenting high in repetitive sequences, hence potentially indicating sequencing biases rather than genetic variability (Figure S8). 

In addition, by acknowledging the relevance of having available a tool for fast and reliable yeast identification in a clinical setting, we evaluated the potential of the PCR-RFLP approach in two well-known clinically relevant situations: the *Candida parapsilosis* complex [35] and the *Aspergillus fumigatus*/*niger*/*flavus*/*terreus* section [36]. Concerning the *Candida parapsilosis* complex, whose principal components encompass *C. parapsilosis*, *C. orthopsilosis*, and *Candida metapsilosis* [35], sets of PCR-RFLP profile combinations were identified to discriminate precisely between these three species: *C. parapsilosis* with HaeIII and StuI, *C. orthopsilosis* with BseSI and NcoI, and *Candida metapsilosis* with BseSI, StyI, and FaiI. Conversely, the *Aspergillus* spp. section, as previously evidenced (Table S2) to show the perfect identity among sequences of some of these species, forbade precise identification. The *A. flavus* species does not show enough variation to allow for precise identification. In particular, the combination of AvaII, CviAII_NlaIII, EsaBC3I_TaqI, and TauI restriction profiles resulted in the identification of *A. fumigatus* with five false positive species: *A. lentulus*, *A. novofumigatus*, *A. oerlinghausenensis*, *A. turcosus*, and *A. udagawae*. The combination of CfoI/HhaI/HinP1I, CviAII/NlaIII, FaiI, and TauI allowed for the identification of *A. niger* together with *A. awamori*. As previously reported in the manuscript, the sequences of *A. flavus*, *A. oryzae*, *A. parasiticus*, and *A. texensis* are the same, hence making it not possible to use this approach for their identification, but the group can be identified from other species with the combination of AvaI, FaiI, SpeI, and TauI. Finally, *A. terreus* can be precisely identified by combining the profiles obtained through AvaI, SetI, and XmaIII digestion.

We have translated all this information into a freely available tool for the identification of yeast species by providing the ITS1-5.8S-ITS2 amplicon length and at least one restriction profile obtained with one or up to five endonucleases included in this study. The tool, named “FId, FungalIdentifier”, is freely accessible (https://stefyeast.shinyapps.io/Fid_app/, last update 22 August 2024) and can also be used to obtain the restriction profiles calculated in silico for all the yeast species included in this study. Upon data entry, the tool outputs the list of species identified as having profiles matching the query profile and also provides relevant information on the characteristics of the species (e.g., isolation sources, phenotypic cellular and colony characteristics, metabolic features).

## 4. Discussion

Recent studies have highlighted the environmental origin of fungi known as a severe threat to human health [37,38] and the potential of natural yeast strains for biotechnological applications [39,40]. To get the best out of this powerful resource, it is fundamental to be able to rapidly screen and identify large numbers of microbial isolates. The revived interest in culturomics approaches for the identification and characterization of yeast isolates from multiple environments has highlighted the need for an update of classical approaches widely used and yet not updated to fulfill and accommodate the improved knowledge on genetic and phenotypic variability. With this study, we have improved and broadened the potential application of PCR-RFLP for the identification of Ascomycete isolates. First of all, we have updated the available database of PCR-RFLP profiles which now includes information on 1461 yeast species (ten-fold more than the number of species included in the previously available database). In addition, the comparison of the performance of 143 different endonucleases in providing PCR-RFLP profiles suitable for yeast identification at the species level allowed us to gather fundamental information that could be exploited for the informed selection of the most appropriate approach according to the predicted yeast species. All this information was fundamental for the development of an open access tool, FId, that will represent a fundamental resource for supporting multiple fields of microbiology, primarily for ecological studies, and easing the high-throughput identification of yeast isolates. The approach and the resulting FId tool have shown, through the evaluation of the performance on experimental data, limits and advantages compared to alternative methods currently adopted for the identification of Ascomycete isolates. While preparing a tool effectively efficient for experimentally gathered data, we paid particular attention to designing a tool amortizing potential (not evitable) experimental/technical errors. The experimental analysis of the restriction patterns can be carried out using horizontal agarose gel electrophoresis or capillary electrophoresis. In the latter case, the precision of the assessment of the DNA fragments can resemble the precision obtained in silico, as it can be used to discriminate molecules differing for a few nucleotides. For instance, it is used for the analysis of short tandem repeats (STRs) [41] or simple sequence repeats (SSRs) [42], consisting of the quantification of the number of repetitions of sequences as short as a couple of nucleotides. However, despite being less precise for the quantification of fragment lengths, horizontal agarose gel electrophoresis is more affordable and currently present in most laboratories studying microbial ecology, compared to capillary electrophoresis. Furthermore, the limited precision of the identification of fungal species can be stemmed by combining multiple endonuclease profiles. This approach was crucial when considering the *Candida parapsilosis* group, including fungi associated with serious human health issues. FId, by using specific combinations of PCR-RFLP profiles, allows for the precise identification of *C. parapsilosis*, *C. orthopsilosis*, and *Candida metapsilosis* species associated with invasive superficial and disseminated infections mostly in neonates and infants [43], and currently identified through a combination of culturomics, microscopic observations, and phenotypic characterizations that have shown issues in providing the reliable and accurate identification of *Candida* isolates at the species level [44,45]. The same results were not achievable in the case of another threat to human health: the *Aspergillus fumigatus*, *A. niger*, *A. flavus*, and *A. terreus* section. In this case, due to the high level of similarity of the ITS1-5.8S-ITS2 genomic regions of the species, the PCR-RFLP approach, even when combining multiple profiles, failed in the identification at the species level, as previously adopted techniques did [46]. To solve this issue, previous studies have identified the need to focus on genomic regions specifically selected for this intent [47].

The validation of the applicability of FId and the PCR-RFLP approach was also performed by comparing the intra-specific variability for highly represented species (e.g., *A. fumigatus*, *A. niger*, *C. albicans*, and *S. cerevisiae*). Besides a few rare cases of genetic variations hindering the possibility of correct identification for some *S. cerevisiae* strains (potentially ascribable to the sequencing of the investigated region), PCR-RFLP and FId allowed for the identification of the correct species, even considering the intra-specific genetic variability. 

By providing, together with the list of species corresponding to the query profile, the environmental, phenotypic, and metabolic characteristics of the identified species, the user will have the possibility of further selecting the potential correct identification according to experimentally gathered data such as cell and colony morphology and source of isolation.

Overall, we trust that the results we provide with this study and the resulting tools will provide resourceful support for advancements in our knowledge on yeast populations and the experimental testing of yeast ecological roles and evolution.

## Data Availability

Genomic data presented in this study are available in NCBI at https://www.ncbi.nlm.nih.gov/datasets/genome/ (accessed on 1 July 2022); reference numbers are reported in Table S1. These data were derived from the following resources available in the public domain, as described in Table S1. Sanger sequencing data presented in this study are available in NCBI at https://www.ncbi.nlm.nih.gov/ (accessed on 1 July 2022); reference numbers are reported in Tables S5 and S6. These data were derived from the following resources available in the public domain, as described in Tables S5 and S6. The original contributions presented in the study are included in the article or in the Supplementary Material; further inquiries can be directed to the corresponding author.

## Supplementary Materials

The following supporting information can be downloaded at: https://www.mdpi.com/article/10.3390/jof10090595/s1, Figure S1: Relationships between the number of observed profiles and identifiable yeast species for all the tested endonucleases; Figure S2: Comparison of identifiable yeast species according to the PCR-RFLP obtained with every tested endonuclease; Figure S3: Percentage of species identifiable according to the combination of PCR-RFLP profiles of two endonucleases; Figure S4: Comparison of in silico and experimental PCR-RFLP results; Figure S5: Comparison of the performance of the lm and the VarPow approaches in the identification of fungal species; Figure S6: Summary of issues affecting the identification of experimentally obtained profiles; Figure S7: Performance of yeast species identification based on two enzyme profiles; Figure S8: WebLogo [48] representation of the intra-specific conservation of the ITS1-5.8S-ITS2 genomic region sequence; Table S1: List of sequences analyzed in this study and in Esteve-Zarzoso et al.’s work; Table S2: ITS1-5.8S-ITS2 amplicon length of the species included in this work; Table S3: Summary of endonuclease performance: Table S4: Details of the results of species identification by using two and three enzyme profiles; Table S5: Experimental data used to train the models for experimental error correction; Table S6: Experimental data used to test the models for identification; Table S7: Details of the results of species identification by using two and three enzyme profiles after the application of the experimental correction lm and VarPow approaches. Table S8: FId results on experimental data. File S1: All the Python and R codes generated to perform the analysis reported in the study.
