# Supplementary material for: Fungal Identifier (FId): An Updated Polymerase Chain Reaction–Restriction Fragment Length Polymorphism Approach to Ease Ascomycetous Yeast Isolates’ Identification in Ecological Studies
Source: J Fungi (Basel). 2024 Aug 23;10(9):595. doi: 10.3390/jof10090595 (PMC11433625; doi:10.3390/jof10090595)
Supplement: Supplementary file 1 [file jof-10-00595-s001.zip › File S1.pdf]

# Fungal Identifier (FId): an updated PCR-RFLP approach to ease Ascomycetous yeast isolates identification in ecological studies

This pdf includes

**Scripts**

definition_of_amplicon_size.py
get_length_of_fragments.py
lmErrFunction.R
powerErrFunction.R
select_on_amplicon_length.R
select_on_digestion.R

## *definition_of_amplicon_size.py*

```
##usage python definition_of_amplicon_size.py <input multifasta> <outfile>

import sys
inputs=sys.argv

InFile=inputs[1]
inputFile=open(InFile)
outFileName=inputs[2]
outFile=open(outFileName,"w")

print("preview of the results: ")
for sequences in inputFile:
    if sequences.startswith(">"):
        name=sequences .strip()[1:]
    else:
        sequence=len(sequences)
        name1=name+"\t"+str(sequence)
        outFile.write(name1+"\n")
```

***back_to_index***

## *get_length_of_fragments.py*

```
##usage python get_length_of_fragments.py <input multifasta> <outfile> <enzyme>

import sys
import re

inputs=sys.argv

InFile=inputs[1]
inputFile=open(InFile)
restrictionEnzyme=inputs[3]
outFileNameBegin=inputs[2]
outFileName=outFileNameBegin+"_"+restrictionEnzyme+".txt"
outFile=open(outFileName,"w")

EnzymesList={"HaeIII":"GGCC","HinfI":"GA(A|T|C|G)TC","CfoI_HhaI_HinP1I":"GCGC",
"AatII_ZraI":"GACGTC", "AbsI":"CCTCGAGG", "Acc65I_Asp718I_KpnI":"GGTACC",
"AccI":"GT(A|C)(T|G)AC", "AceII_BmtI_NheI":"GCTAGC", "AciI":"(CCGC|GCGG)",
"AclI":"AACGTT", "AcyI":"G(A|G)CG(C|T)C", "AfeI_Eco47III":"AGCGCT",
"AflII":"CTTAAG", "AflIII":"AC(A|G)(C|T)GT", "AgeI":"ACCGGT", "AgsI":"TT(G|
C)AA", "AhaIII_DraI":"TTTAAA","AluI":"AGCT", "ApaI_Bsp120I_PspOMI":"GGGCCC",
"ApaLI":"GTGCAC", "ApoI":"(A|G)AATT(C|T)", "AscI":"GGCGCGCC",
"AseI_VspI":"ATTAAT","Asi256I_BspKT6I_ChaI_DpnI":"GATC",
"AsiSI_SgfI":"GCGATCGC", "AsuII_BstBI":"TTCGAA", "AvaI":"C(C|T)CG(A|G)G",
"AvaII":"GG(A|T)CC","AvrII":"CCTAGG", "BalI":"TGGCCA", "BamHI":"GGATCC",
"BbeI_Eco78I_KasI_NarI_PluTI_SfoI":"GGCGCC", "BbsI":"GAAGAC", "BclI":"TGATCA",
"BetI":"(A|T)CCGG(A|T)","BfaI_MaeI":"CTAG", "BglII":"AGATCT",
"BmgBI_BtrI_PmaCI_PmlI":"CACGTG", "Bpu10I":"(CCT(A|T|C|G)AGC)|(GCT(A|T|C|
G)AGG)", "BsaAI":"(C|T)ACGT(A|G)", "BsePI_BssHII":"GCGCGC","BseSI":"G(T|G)GC(A|
C)C", "BseYI":"(CCCAGC)|(GCTGGG)", "BssSI-v2_Nb.BssSI":"(CACGAG)|(CTCGTG)",
"BsiWI":"CGTACG", "Bsp1407I":"TGTACA", "BspEI_BspMII":"TCCGGA","BspHI":"TCATGA",
"BspLU11I_PciI":"ACATGT",  "BsrBI":"(CCGCTC)|(GAGCGG)","BstEII":"GGT(A|T|C|
G)ACC", "BstNI_EcoRII":"CC(A|T)GG", "BstUI_FnuDII":"CGCG", "Cac8I":"GC(A|T|C|G)
(A|T|C|G)GC", "CauII":"CC(G|C)GG","CdiI":"(CATCG)|(CGATG)", "Cfr10I":"(A|
G)CCGG(C|T)", "CfrI":"(C|T)GGCC(A|G)", "ClaI":"ATCGAT", "CviAII_NlaIII":"CATG",
"CviQI_RsaI":"GTAC", "CviRI_HpyCH4V":"TGCA", "DdeI":"CT(A|T|C|G)AG","DraII":"(A|
G)GG(A|T|C|G)CC(C|T)", "Eco53kI_EcoICRI_SacI":"GAGCTC",
"Eco56I_NaeI_NgoMIV":"GCCGGC", "EcoRI":"GAATTC", "EcoRV":"GATATC",
"EcoT22I_NsiI_Ppu10I":"ATGCAT", "EsaBC3I_TaqI":"TCGA", "FaiI":"(C|T)AT(A|G)",
"Fnu4HI":"GC(A|T|C|G)GC","FseI":"GGCCGGCC", "FspAI":"(A|G)TGCGCA(C|T)",
"FspI":"TGCGCA", "HaeII":"(A|G)GCGC(C|T)", "HgiJII":"G(A|G)GC(C|T)C",
"HindII":"GT(C|T)(A|G)AC", "HindIII":"AAGCTT","HpaI":"GTTAAC",
"HpaII_MspI":"CCGG", "Hpy178III":"TC(A|T|C|G)(A|T|C|G)GA", "Hpy188I":"TC(A|T|C|
G)GA", "Hpy8I":"GT(A|T|C|G)(A|T|C|G)AC", "Hpy99I":"CG(A|T)CG",
"HpyCH4IV_MaeII_TaiI":"ACGT", "I-CeuI":"(TAACTATAACGGTCCTAAGGTAGCGAA)|
(TTCGCTACCTTAGGACCGTTATAGTTA)", "I-SceI":"(TAGGGATAACAGGGTAAT)|
(ATTACCCTGTTATCCCTA)", "LmnI":"(GCTCC(A|T|C|G))|((A|T|C|G)GGAGC)",
 "MauBI":"CGCGCGCG", "MfeI":"CAATTG", "MluI":"ACGCGT", "MseI":"TTAA",
"Nb.BbvCI_Nt.BbvCI":"(CCTCAGC)|(GCTGAGG)", "Nb.BsmI":"(GAATGC(A|T|C|G))|((A|T|C|
G)GCATTC)", "Nb.BsrDI":"(GCAATG(A|T|C|G)(A|T|C|G))|((A|T|C|G)(A|T|C|G)CATTGC)",
"Nb.BtsI":"(GCAGTG(A|T|C|G)(A|T|C|G))|((A|T|C|G)(A|T|C|G)CACTGC)",
"NcoI":"CCATGG", "NdeI":"CATATG","NlaIV":"GG(A|T|C|G)(A|T|C|G)CC",
"NotI":"GCGGCCGC", "NruI":"TCGCGA",  "NspI":"(A|G)CATG(C|T)",
"PacI":"TTAATTAA", "PasI":"CCC(A|T)GGG", "PfoI":"TCC(A|T|C|G)GGA", "PI-
PspI":"(TGGCAAACAGCTATTATGGGTATTATGGGT)|(ACCCATAATACCCATAATAGCTGTTTGCCA)", "PI-
SceI":"(ATCTATGTCGGGTGCGGAGAAAGAGGTAAT)|(ATTACCTCTTTCTCCGCACCCGACATAGAT)",
"PmeI":"GTTTAAAC", "PpuMI":"(A|G)GG(A|T)CC(C|T)", "PshAI":"GAC(A|T|C|G)(A|T|C|G)
(A|T|C|G)(A|T|C|G)GTC", "PsiI":"TTATAA","PspXI":"(A|C|G)CTCGAG(T|C|G)",
"PstI":"CTGCAG", "PvuI":"CGATCG", "PvuII":"CAGCTG", "RsrII":"CGG(A|T)CCG",
"SacII":"CCGCGG", "SalI":"GTCGAC","SanDI":"GGG(A|T)CCC",
"SbfI_Sse8387I":"CCTGCAGG", "ScaI":"AGTACT", "ScrFI":"CC(A|T|C|G)GG",
```

```
"SduI":"G(A|T|G)GC(A|T|C)C", "SetI":"A(G|C)(G|C)T", "SexAI":"ACC(A|
T)GGT","SgrAI":"C(A|G)CCGG(C|T)G", "SgrDI":"CGTCGACG", "SimI":"(GGGTC)|(GACCC)",
"SmaI_XmaI":"CCCGGG", "SmlI":"CT(C|T)(A|G)AG", "SnaBI":"TACGTA",
"SpeI":"ACTAGT", "SphI":"GCATGC", "SrfI":"GCCCGGGC",
"Sse232I":"CGCCGGCG","SspI":"AATATT", "StuI":"AGGCCT", "StyI":"CC(A|T)(A|T)GG",
"SwaI":"ATTTAAAT", "TatI":"(A|T)GTAC(A|T)", "TauI":"GC(G|C)GC", "TfiI":"GA(A|
T)TC","TseI":"GC(A|T)GC",  "XbaI":"TCTAGA", "XhoI":"CTCGAG", "XhoII":"(A|
G)GATC(C|T)", "XmaIII":"CGGCCG"}

CutEnzymes={"HaeIII":"2","HinfI":"1","CfoI_HhaI_HinP1I":"3","AatII_ZraI":"5",
"AbsI":"2", "Acc65I_Asp718I_KpnI":"1", "AccI":"2", "AceII_BmtI_NheI":"5",
"AciI":"1", "AclI":"2", "AcyI":"2", "AfeI_Eco47III":"3", "AflII":"1",
"AflIII":"1", "AgeI":"1", "AgsI":"3", "AhaIII_DraI":"3", "AluI":"2",
"ApaI_Bsp120I_PspOMI":"5", "ApaLI":"1", "ApoI":"1", "AscI":"2", "AseI_VspI":"2",
"Asi256I_BspKT6I_ChaI_DpnI":"1", "AsiSI_SgfI":"5", "AsuII_BstBI":"2",
"AvaI":"1", "AvaII":"1", "AvrII":"1", "BalI":"3", "BamHI":"1",
"BbeI_Eco78I_KasI_NarI_PluTI_SfoI":"5","BbsI":"6", "BclI":"1", "BetI":"1",
"BfaI_MaeI":"1", "BglII":"1", "BmgBI_BtrI_PmaCI_PmlI":"3", "Bpu10I":"2",
"BsaAI":"2", "BsePI_BssHII":"1", "BseSI":"5", "BseYI":"1", "BssSI-
v2_Nb.BssSI":"1", "BsiWI":"1","Bsp1407I":"1", "BspEI_BspMII":"1", "BspHI":"1",
"BspLU11I_PciI":"1","BsrBI":"3","BstEII":"1","BstNI_EcoRII":"2","BstUI_FnuDII":"
2", "Cac8I":"3", "CauII":"1", "CdiI":"4", "Cfr10I":"1", "CfrI":"3", "ClaI":"2",
"CviAII_NlaIII":"1", "CviQI_RsaI":"1", "CviRI_HpyCH4V":"2", "DdeI":"1",
"DraII":"2", "Eco53kI_EcoICRI_SacI":"3", "Eco56I_NaeI_NgoMIV":"1", "EcoRI":"1",
"EcoRV":"3", "EcoT22I_NsiI_Ppu10I":"5", "EsaBC3I_TaqI":"2", "FaiI":"2",
"Fnu4HI":"2","FseI":"6", "FspAI":"4", "FspI":"3", "HaeII":"5", "HgiJII":"5",
"HindII":"3", "HindIII":"1", "HpaI":"3", "HpaII_MspI":"1","Hpy178III":"2",
"Hpy188I":"3", "Hpy8I":"3", "Hpy99I":"5", "HpyCH4IV_MaeII_TaiI":"1", "I-
CeuI":"17", "I-SceI":"9", "LmnI":"5","MauBI":"2", "MfeI":"1", "MluI":"1",
"MseI":"1", "Nb.BbvCI_Nt.BbvCI":"5", "Nb.BsmI":"5", "Nb.BsrDI":"6",
"Nb.BtsI":"6", "NcoI":"1", "NdeI":"2", "NlaIV":"3", "NotI":"2", "NruI":"3",
"NspI":"5", "PacI":"5", "PasI":"2", "PfoI":"1", "PI-PspI":"17", "PI-SceI":"15",
"PmeI":"4", "PpuMI":"2", "PshAI":"6", "PsiI":"3","PspXI":"2", "PstI":"5",
"PvuI":"4", "PvuII":"3", "RsrII":"2", "SacII":"4", "SalI":"1", "SanDI":"2",
"SbfI_Sse8387I":"6", "ScaI":"3", "ScrFI":"2", "SduI":"5","SetI":"4",
"SexAI":"1", "SgrAI":"2", "SgrDI":"2", "SimI":"2", "SmaI_XmaI":"3", "SmlI":"1",
"SnaBI":"3", "SpeI":"1", "SphI":"5", "SrfI":"4", "Sse232I":"2","SspI":"3",
"StuI":"3", "StyI":"1", "SwaI":"4", "TatI":"1", "TauI":"4", "TfiI":"1",
"TseI":"1", "XbaI":"1", "XhoI":"1", "XhoII":"1","XmaIII":"1"}


sequenceTarget=EnzymesList[restrictionEnzyme]
print(sequenceTarget)
lengthBlunt=CutEnzymes[restrictionEnzyme]
outLine=""
print("preview of the results: ")
for sequences in inputFile:
    if sequences.startswith(">"):
        name=sequences.strip()
    else:
        sequence=sequences.upper()
        if not restrictionEnzyme in ["HinfI", "AccI", "AciI", "AcyI", "AflIII",
"AgsI", "ApoI", "AvaI", "AvaII", "BetI", "Bpu10I", "BsaAI", "BseSI", "BseYI",
"BssSI-v2_Nb.BssSI", "BsrBI", "BstEII", "BstNI_EcoRII", "Cac8I", "CauII",
"CdiI", "Cfr10I", "CfrI", "DdeI", "DraII", "FaiI", "Fnu4HI", "FspAI", "HaeII",
"HgiJII", "HindII", "Hpy178III","Hpy188I", "Hpy8I", "Hpy99I", "I-CeuI", "I-
SceI", "LmnI",  "Nb.BbvCI_Nt.BbvCI", "Nb.BsmI", "Nb.BsrDI", "Nb.BtsI", "NlaIV",
"NspI", "PasI", "PfoI", "PI-PspI", "PI-SceI", "PpuMI", "PshAI", "PspXI",
"RsrII", "SanDI", "ScrFI", "SduI", "SetI", "SexAI", "SgrAI", "SimI", "SmlI",
"StyI", "TatI", "TauI", "TfiI", "TseI", "XhoII"]:
            fragment=sequence.find(sequenceTarget)
            1stFragment=sequence[:fragment+int(lengthBlunt)]
            remainance=sequence[fragment+int(lengthBlunt):]
            if not fragment==-1:
```

```
        outLine=name[1:]+"\t"+str(fragment+int(lengthBlunt))
    else:
        outLine=name[1:]+"\t"+str(len(sequence)-1)
    while not fragment==-1:
        second=remainance.find(sequenceTarget)
        remainance=remainance[second+int(lengthBlunt):]
        if not second==-1:
            outLine=outLine+"\t"+str(second+int(lengthBlunt))
        else:
            outLine=outLine+"\t"+str(len(remainance)-1)
        fragment=second
else:
    if re.search(sequenceTarget,sequence):
        fragment1=re.search(sequenceTarget,sequence)
        fragment=sequence.find(fragment1.group(0))
        1stFragment=sequence[:fragment+int(lengthBlunt)]
        remainance=sequence[fragment+int(lengthBlunt):]
        outLine=name[1:]+"\t"+str(fragment+int(lengthBlunt))
        while re.search(sequenceTarget,remainance):
            second1=re.search(sequenceTarget,remainance)
            second=remainance.find(second1.group(0))
            remainance=remainance[second+int(lengthBlunt):]
            outLine=outLine+"\t"+str(second+int(lengthBlunt))
        outLine=outLine+"\t"+str(len(remainance)-1)
    else:
        outLine=name[1:]+"\t"+str(len(sequence)-1)
outFile.write(outLine+"\n")
```

***back_to_index***

## lmErrFunction.R

```
lmErrFunction=function(input_number){
  inputExpUpp=round(((as.numeric(m.0$coefficients[2])*input_number+as.numeric(m.
       0$coefficients[1]))+2*m.0$sigma*sqrt(((1307*(input_number)^2)-
       (2*A*input_number+B)/denom),2)
  inputExpLow=round(((as.numeric(m.0$coefficients[2])*input_number+as.numeric(m.
       0$coefficients[1]))-2*m.0$sigma*sqrt(((1307*(input_number)^2)-
       (2*A*input_number+B)/denom),2)
  output=list(inputExpFit,inputExpLow,inputExpUpp)
  names(output)=c("fit","lower","upper")
  return(output)
}
```

back_to_index

## powerErrFunction.R

```
powerErrFunction=function(input_number){
  inputExpFit=round(as.numeric(m.0w$coefficients[2])*input_number+as.numeric(m.0
       w$coefficients[1]),2)
  inputExpUpp=round((as.numeric(m.0w$coefficients[2])*input_number+as.numeric(m.
       0w$coefficients[1])+2*((as.numeric(powerRes$coefficients[2])*input_number)
       +as.numeric(powerRes$coefficients[1]))),2)
  inputExpLow=round((as.numeric(m.0w$coefficients[2])*input_number+as.numeric(m.
       0w$coefficients[1]))
       +2*((as.numeric(powerRes$coefficients[2])*input_number)
       +as.numeric(powerRes$coefficients[1]))),2)
    output=list(inputExpFit,inputExpLow,inputExpUpp)
    names(output)=c("fit","lower","upper")
    return(output)
}
```

back_to_index

## select_on_amplicon_length.R

```
select_on_amplicon_length=function(reference,inputExp,model=c("lm","VarPow")){
  if (missing(model)|!model%in%c("lm","VarPow")) model="VarPow"
  referenceTable=read.table(reference, header=F,sep="\t")
  colnames(referenceTable)=c("species","amplicon_size")
  if(model=="lm"){
    temp0=lmErrFunctions(as.numeric(inputExp))
  }else if (model=="VarPow"){
    temp0=powerErrFunctions(as.numeric(inputExp))
  }
  inputExpFit=temp0$fit
  inputExpUpp=temp0$upper
  inputExpLow=temp0$lower

referenceSel=referenceTable[as.numeric(referenceTable$amplicon_size)>=as.numeric
(inputExpLow)&

as.numeric(referenceTable$amplicon_size)<=as.numeric(inputExpUpp),]
  nmatches=nrow(referenceSel)
  output=list(referenceSel,nmatches)
  names(output)=c("selected_profiles","n_of_matches")
  return(output)
}
```

back_to_index

### select_on_digestion.R

```
select_on_digestion=function(input,referenceSel,listSelAmpl,model=c("lm","VarPow
")){
  # input= the profile of digestion, written as i.e. "536+218+150"
  # referenceSel = the path to the file with the results of enzyme digestion
  # listSelAmpl=listSelAmpl$selected_profiles$species, output of
select_on_amplicon_length.R
  if (missing(model)|!model%in%c("lm","VarPow")) model="VarPow"
  referenceEnzTable=read.table(referenceSel, header=F,sep="\t",row.names=1)
  EnzChosen=referenceEnzTable[row.names(referenceEnzTable)%in
%listSelAmpl$species,]
  if(is.data.frame(EnzChosen)){
    EnzChosen1=t(apply(EnzChosen,1,function(x) sort(x,decreasing = T)))
    EnzChosen1[EnzChosen1<100]=0
    EnzChosen1=EnzChosen1[,colSums(EnzChosen1)!=0]
  }else{EnzChosen1=EnzChosen}
  inputVals=unlist(strsplit(input,split="[+]"))
  if(is.matrix(EnzChosen1)){
    summaryResults=matrix(0,nrow=nrow(EnzChosen1),ncol=2)
    row.names(summaryResults)=row.names(EnzChosen1)
    colnames(summaryResults)=c("matching_fragments","expected_fragments")
    summaryResults[,2]=apply(EnzChosen1,1,function(x) length(x[x!=0]))
  }else{
    summaryResults=matrix(0,nrow=length(EnzChosen1),ncol=2)
    row.names(summaryResults)=names(EnzChosen1)
    colnames(summaryResults)=c("matching_fragments","expected_fragments")
    summaryResults[,2]=length(EnzChosen1[EnzChosen1!=0])
  }
  for(fragments in 1:length(inputVals)){
    if(model=="lm"){
      temp0=lmErrFunctions(as.numeric(inputVals[fragments]))
    }else if (model=="VarPow"){
      temp0=powerErrFunctions(as.numeric(inputVals[fragments]))
    }
    inputExpFit=temp0$fit
    inputExpUpp=temp0$upper
    inputExpLow=temp0$lower
    if(inputExpLow>100){
      if(is.matrix(EnzChosen1)){
        tempSum=t(apply(EnzChosen1,1,function(x)
ifelse(x>=inputExpLow&x<=inputExpUpp,+1,+0)))
summaryResults[,1]=summaryResults[,1]+as.numeric(ifelse(rowSums(tempSum)>=1,1,0)
)
      }else{
        tempSum=ifelse(EnzChosen1>=inputExpLow&EnzChosen1<=inputExpUpp,+1,+0)
summaryResults[,1]=summaryResults[,1]+as.numeric(ifelse(sum(tempSum)>=1,1,0))
      }
    }
  }
  summaryResults=as.data.frame(summaryResults)
  summaryResults$perc=summaryResults[,1]/summaryResults[,2]
  summaryResults1=summaryResults[summaryResults$perc<=1&summaryResults$perc!=0,]
  summaryResults1=summaryResults1[(summaryResults1$matching_fragments/
length(inputVals))==max((summaryResults1$matching_fragments/
length(inputVals))),]
  return((summaryResults1))
}
```

back_to_index
