# Supplementary material for: Fungal Identifier (FId): An Updated Polymerase Chain Reaction–Restriction Fragment Length Polymorphism Approach to Ease Ascomycetous Yeast Isolates’ Identification in Ecological Studies
Source: J Fungi (Basel). 2024 Aug 23;10(9):595. doi: 10.3390/jof10090595 (PMC11433625; doi:10.3390/jof10090595)
Supplement: Supplementary file 1 [file jof-10-00595-s001.zip › Supplementary materials-figures.pdf]

Supplementary materials
for

Fungal Identifier (FId): an updated PCR-RFLP approach to ease yeast isolates identification in the metagenomic era

This pdf includes

Figures

Supplementary Figure S1: Relationships between the number of observed profiles and identifiable yeast species for all the tested endonucleases.
Supplementary Figure S2: Comparison of identifiable yeast species according to the PCR-RFLP obtained with every tested endonuclease.
Supplementary Figure S3: Percentage of species identifiable according to the combination of PCR-RFLP profiles of two endonucleases.
Supplementary Figure S4: Comparison of in silico and experimental PCR-RFLP results.
Supplementary Figure S5: Comparison of the performance of the lm and the VarPow approaches in the identification of fungal species.
Supplementary Figure S6: Summary of issues affecting the identification of experimentally obtained profiles.
Supplementary Figure S7: Performance of yeast species identification based on two enzyme profiles.
Supplementary Figure S8: Intra-specific conservation of the ITS1-5.8S-ITS2 genomic region sequence.

Tables

Supplementary Table S1: List of sequences analyzed in this study and in Esteve-Zarzoso et al. Work.
Supplementary Table S2: ITS1-5.8S-ITS2 amplicon length of the species included in this work.
Supplementary Table S3: Summary of endonuclase performance.
Supplementary Table S4: Presence/absence of restriction sites in the ITS1-5.8S-ITS2 sequence of the species included in the study.
Supplementary Table S5: Details on the results of species identification by using two and three enzymes profiles.
Supplementary Table S6: Experimental data used to train the models for experimental error correction.
Supplementary Table S7: Details on the results of species identification by using two and three enzymes profiles after the application of the experimental correction lm and VarPow approaches.
Table S8: FId results on experimental data.

Scripts

definition_of_amplicon_size.py

Supplementary information

get_length_of_fragments.py
lmErrFunction.R
powerErrFunction.R
select_on_amplicon_length.R
select_on_digestion.R

Supplementary information

Supplementary Figure S1

Relationships between the number of observed profiles and identifiable yeast species for all the tested endonucleases. Each point corresponds to the results of a different endonuclease; the black line indicates the 1:1 correspondence of profiles and identifiable species; the blue line indicates the best fitting of linear regression on data, the formula is reported in blue.

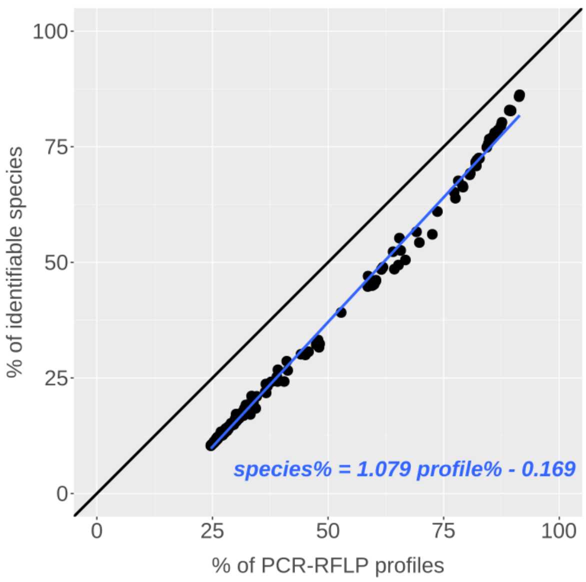

Supplementary information

Supplementary Figure S2

Comparison of identifiable yeast species according to the PCR-RFLP obtained with every tested endonuclease.

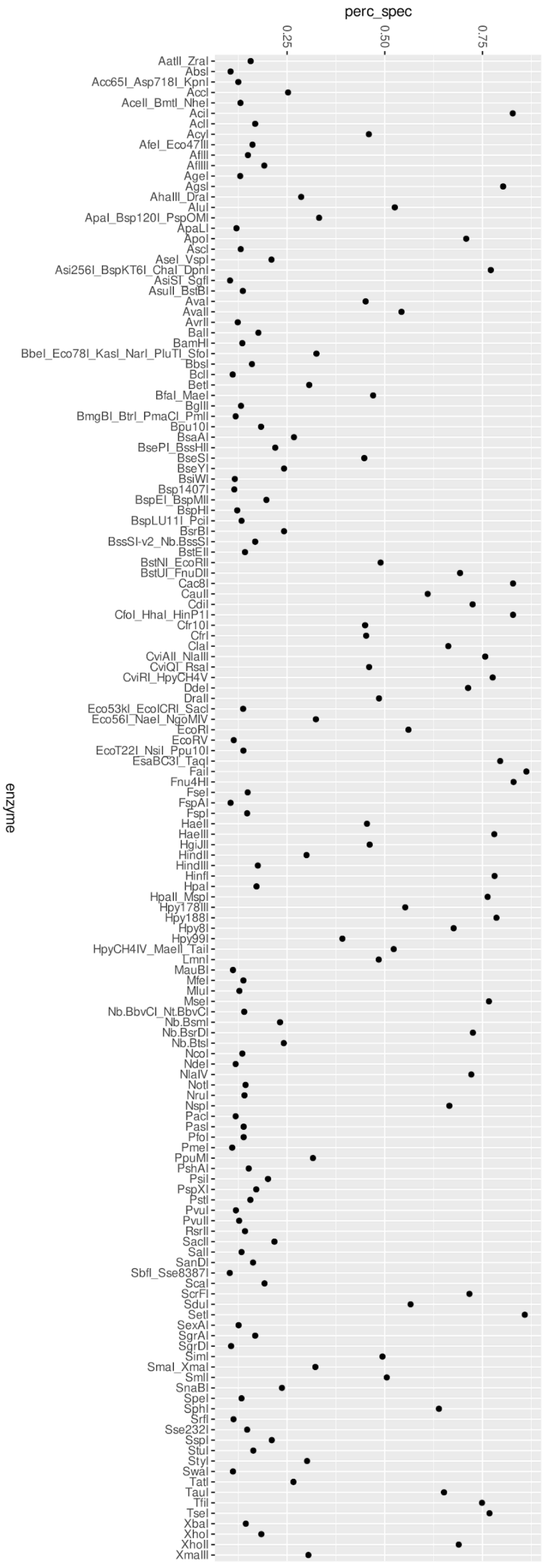

Supplementary Figure S3

Percentage of species identifiable according to the combination of PCR-RFLP profiles of two endonucleases. The color of the heatmap cells indicates the number of yeast species identifiable thanks to the combination of the profiles obtained with the endonucleases indicated in the corresponding row and column.

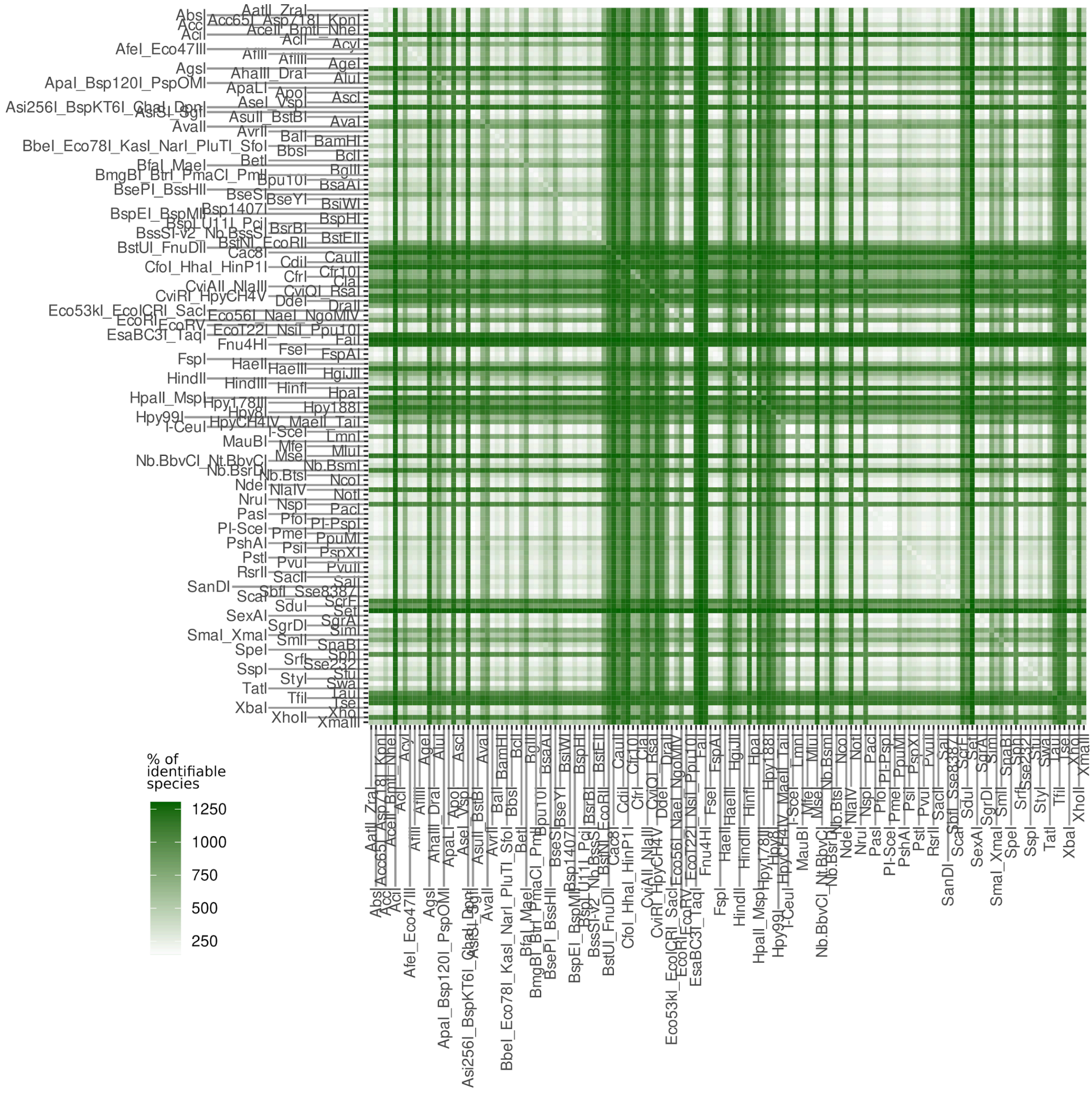

Supplementary information

Supplementary Figure S4: Comparison of *in silico* and experimental PCR-RFLP results.
a) Error percentage of experimental fragment length definition; the error was calculated as the absolute value of the difference of the fragment length assessed *in silico* and experimentally divided by the expected fragment length. The vertical red line indicates 100 bp. **b)** Comparison of the length of fragments obtained experimentally (measured) and *in silico* (expected) and superimposition of the regression lines and relative confidence intervals calculated with the lm and the VarPow approaches (described in materials and methods). c) Summary of the fitting results obtained on a reference experimental dataset with the lm and VarPow approaches.

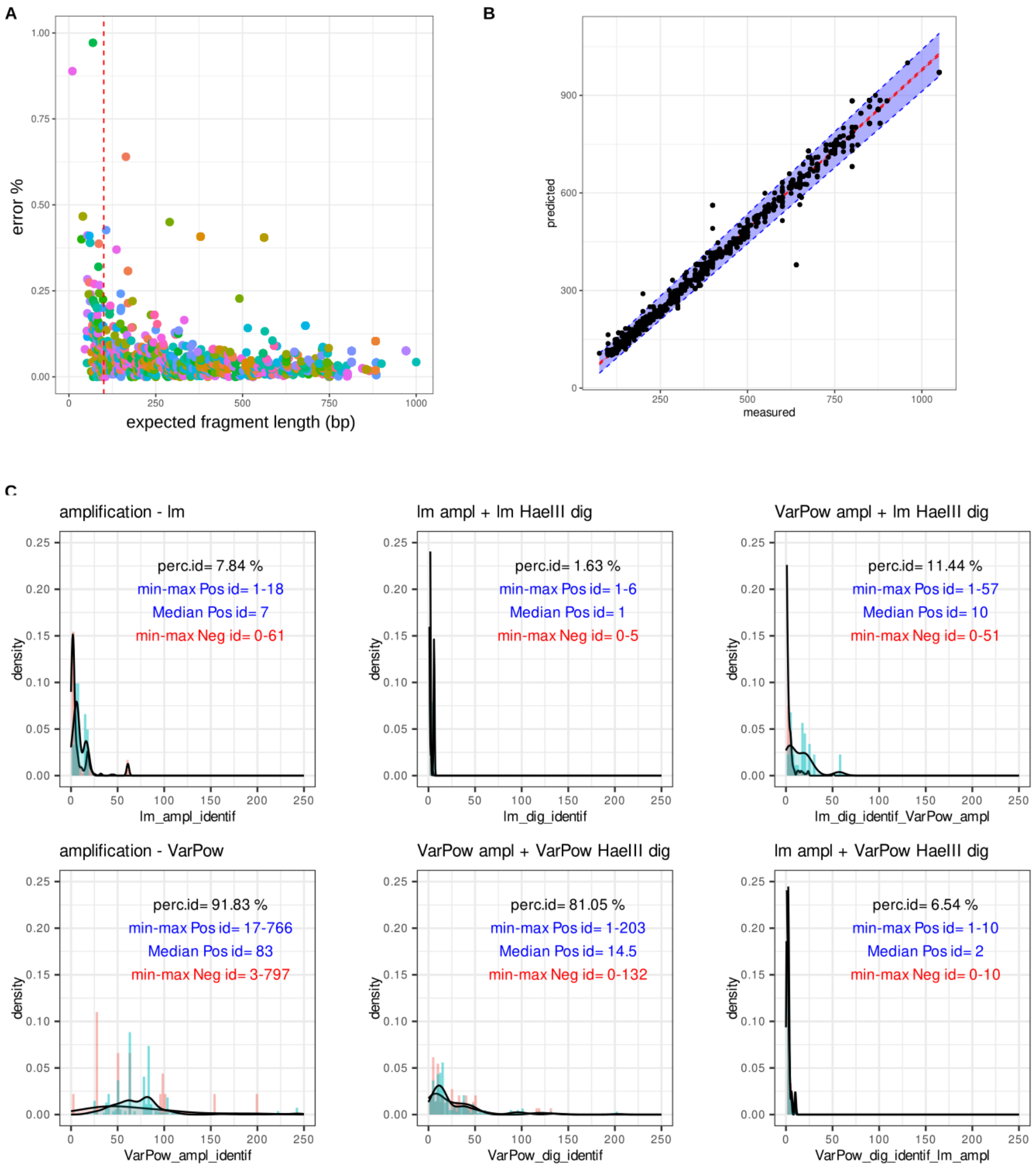

Supplementary information

Supplementary figure S5:
Comparison of the performance of the Im and the VarPow approaches in the identification of fungal species. 100 species were randomly selected and their identification was assessed by using the Im and VarPow approaches on both the amplicon length and the restriction pattern of every investigated endonuclease. Identifications were obtained with the VarPow **(A)** and Im **(B)** approach on amplicon length and successive identification based on the restriction profiles with either Im or VarPow approaches. Color codes are shown in the figure legend, red boxes indicate enzyme allowing the identification of all the tested yeast species. The number of false positives (species identified as potentially matching the query information) resulting with the Im **(C)** and VarPow **(D)** approach on amplicon length and successive identification based on the restriction profiles with either Im or VarPow approaches.

a

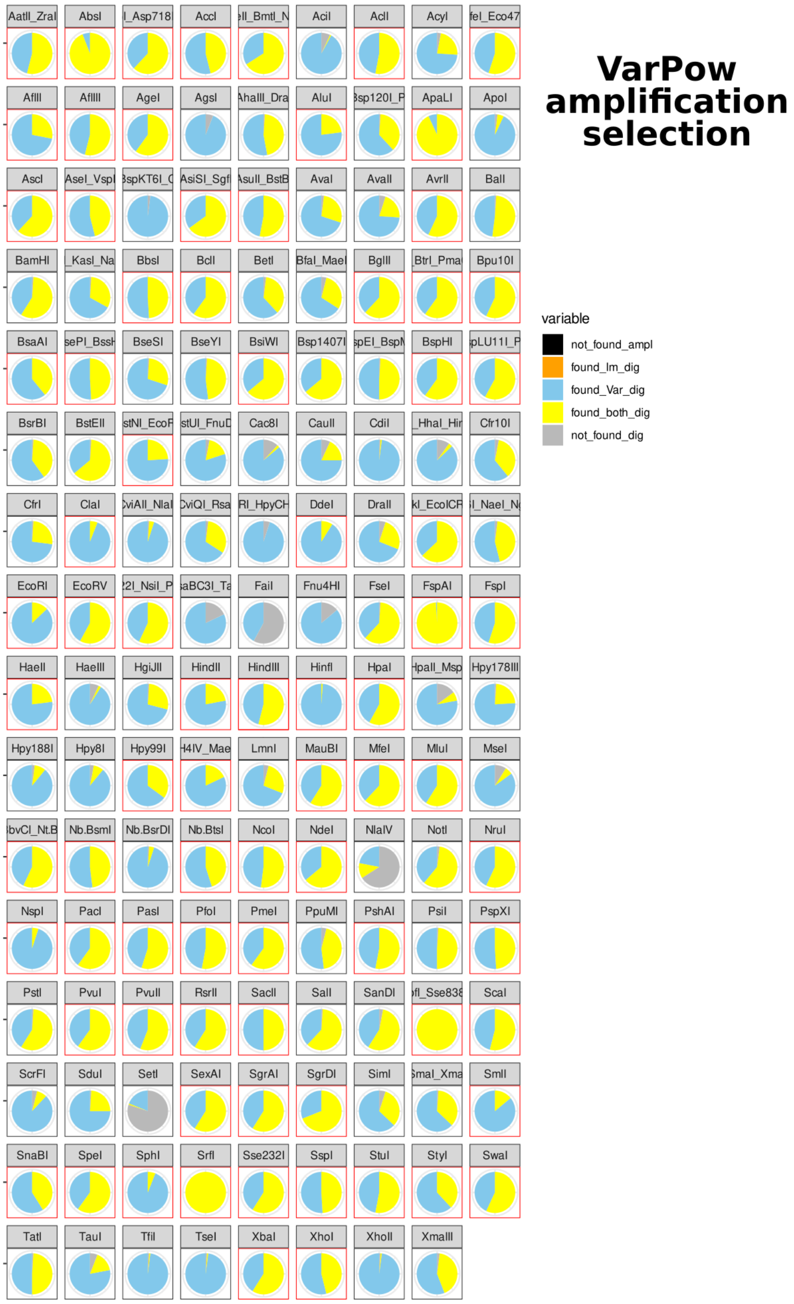

b

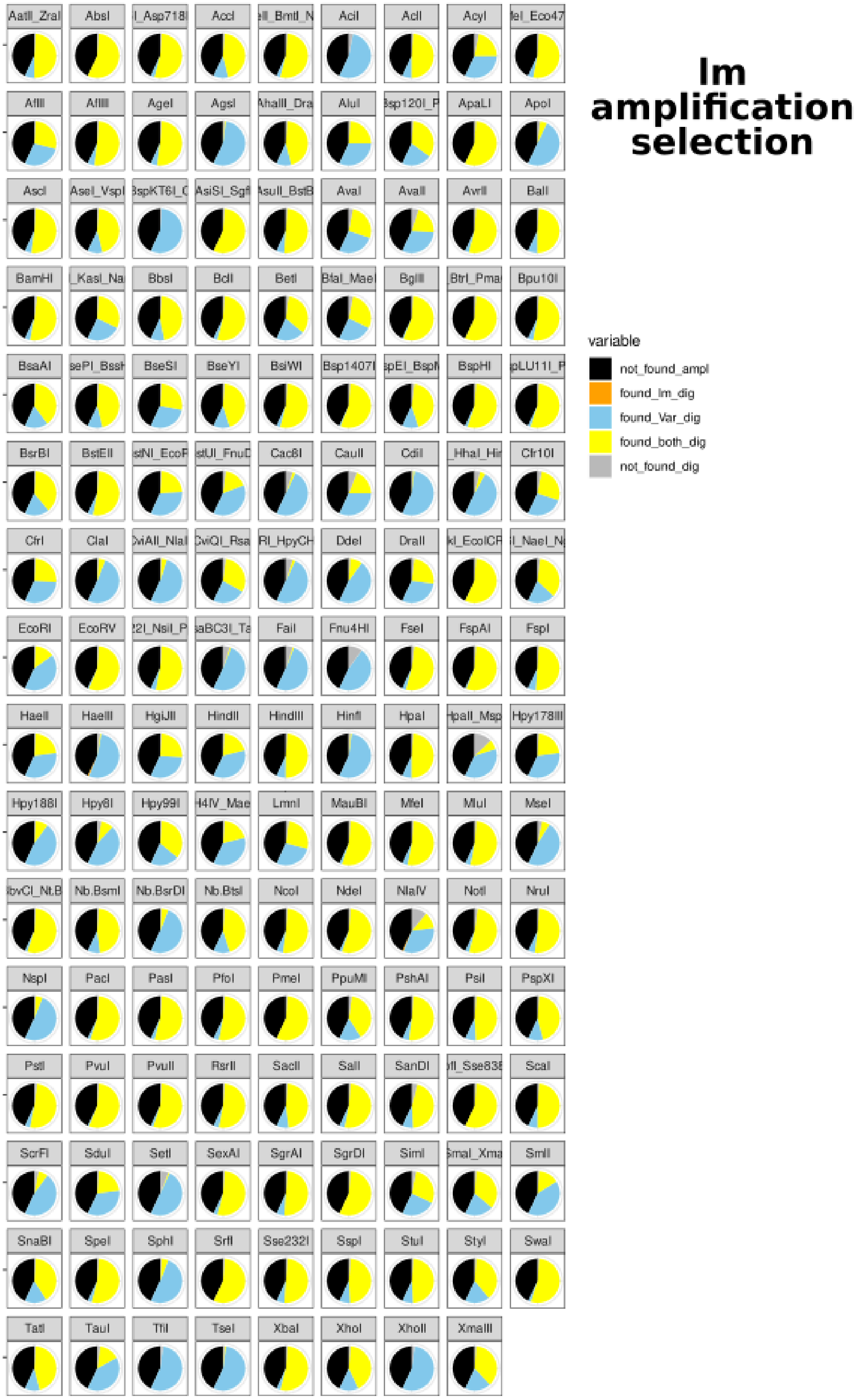
lm amplification selection
variable
not_found_ampl
found_lm_dig
found_Var_dig
found_both_dig
not_found_dig
AatII_ZraI
AbsI
I_Asp718I
AccI
II_BmtI_N
AciI
AclI
AcyI
eI_Eco47
AflII
AflIII
AgeI
AgsI
AhaIII_Dra
AluI
3sp120I_P
ApaLI
ApoI
AscI
AseI_VspI
3spKT6I_C
AsiSI_SgfI
AsuII_BstB
AvaI
AvaII
AvrII
BalI
BamHI
_KasI_Na
BbsI
BclI
BetI
BfaI_MaeI
BglII
BtrI_PmaI
Bpu10I
BsaAI
sePI_BssH
BseSI
BseYI
BsiWI
Bsp1407I
spEI_BspM
BspHI
pLU11I_P
BsrBI
BstEII
stNI_EcoR
stUI_FnuD
Cac8I
CauII
CdiI
_HhaI_Hi
Cfr10I
CfrI
ClaI
CviAII_NlaI
CviQI_Rsa
RI_HpyCH
DdeI
DraII
kI_EcoICR
I_NaeI_N
EcoRI
EcoRV
22I_NsiI_P
aBC3I_Ta
FaiI
Fnu4HI
FseI
FspAI
FspI
HaeII
HaeIII
HgiJII
HindII
HindIII
HinfI
HpaI
HpaII_Msp
Hpy178III
Hpy188I
Hpy8I
Hpy99I
H4IV_Mae
LmnI
MauBI
MfeI
MluI
MseI
bvCI_Nt.B
Nb.BsmI
Nb.BsrDI
Nb.BtsI
NcoI
NdeI
NlaIV
NotI
NruI
NspI
PacI
PasI
PfoI
PmeI
PpuMI
PshAI
PsiI
PspXI
PstI
PvuI
PvuII
RsrII
SacI
SalI
SanDI
oII_Sse838
ScaI
ScrFI
SduI
SetI
SexAI
SgrAI
SgrDI
SimI
SmaI_Xma
SmlI
SnaBI
SpeI
SphI
SrfI
Sse232I
SspI
StuI
StyI
SwaI
TatI
TauI
TfiI
TseI
XbaI
XhoI
XhoII
XmaIII

C

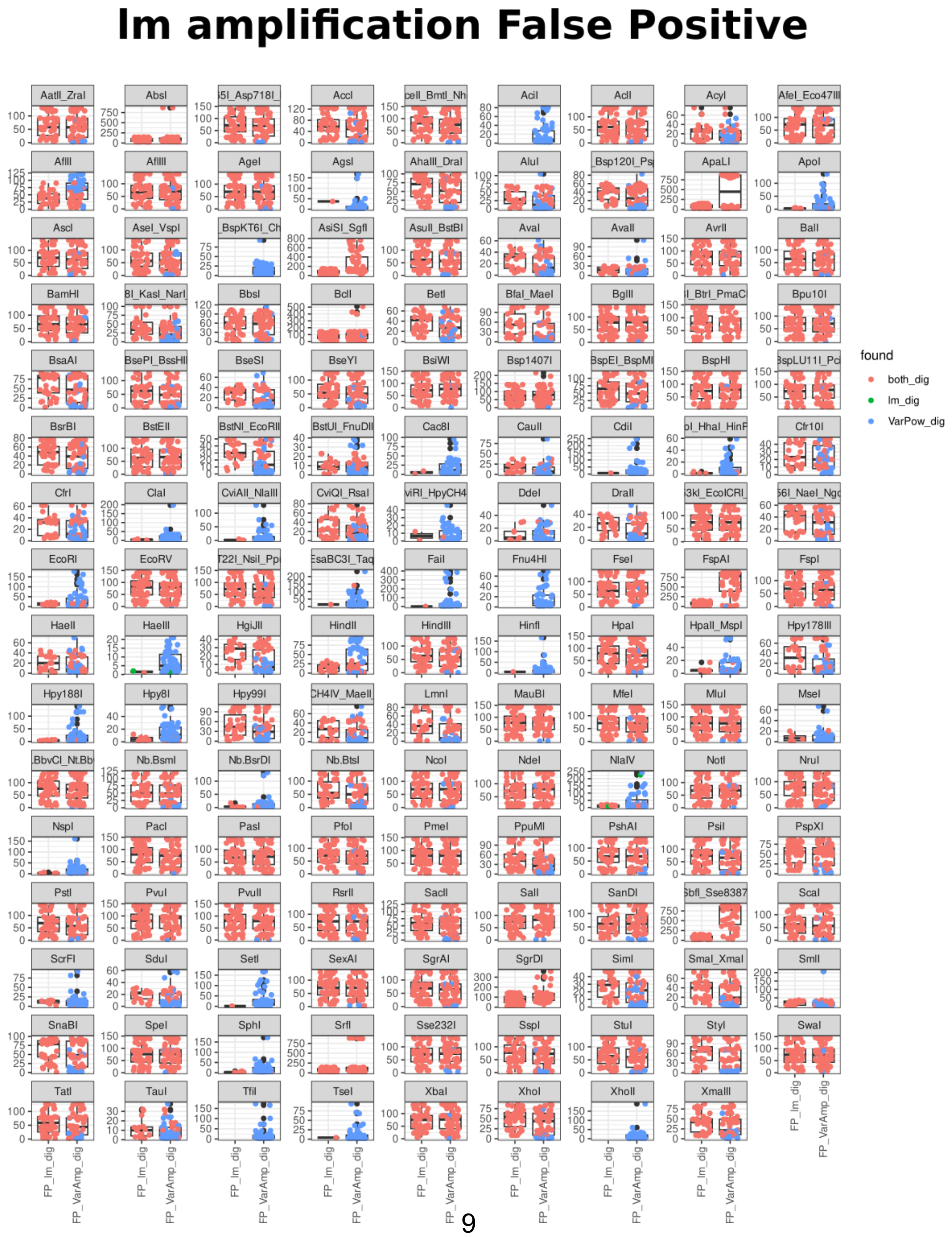
Im amplification False Positive
found
both_dig
Im_dig
VarPow_dig
FP_Im_dig
FP_VarAmp_dig

D

VarPow amplification False Positive

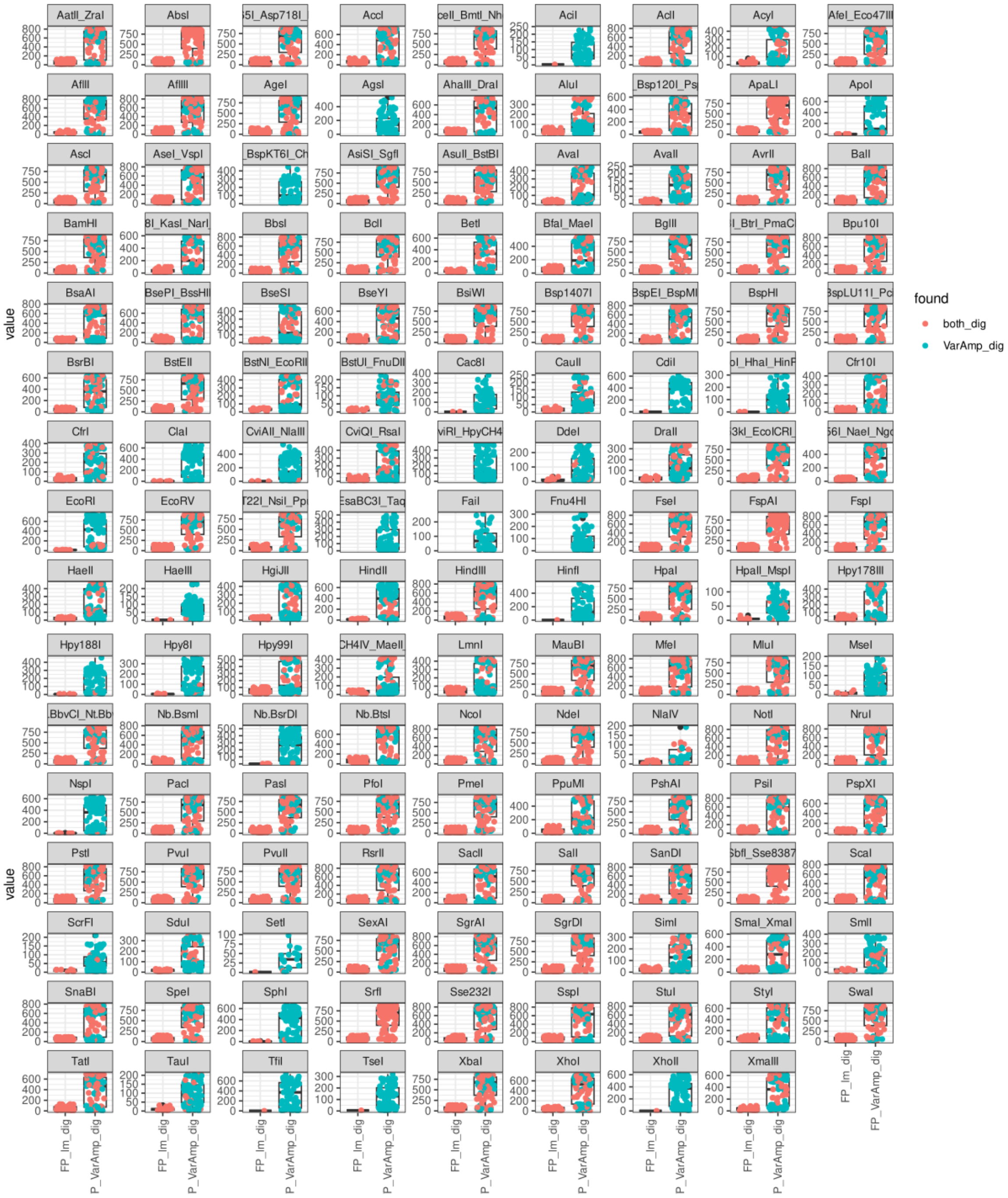

Supplementary information

Supplementary Figure S6:
Summary of issues affecting the identification of experimentally obtained profiles. **a)** Comparison of amplicon lengths associated with yeast species identified or not identified according to the amplicon length analyzed with the lm approach. *= Wilcox-Mann-Withney p-value<0.001. **b)** Fragments shorter than 100 bp are not included in the analysis with experimental data, as the electrophoretic approach does not allow a reliable quantification of the fragment size. The percentage of fragments shorter than 100 bp found in all the 1462 species analyzed is indicated, for each enzyme, in the top left of the plot; the percentage of fragments longer or equal to 100 bp is reported on the top right of the plot. FaiI, SetI, Fnu4HI, and NlaIV are the four enzymes with the lowest percentage of identified yeast species according both to the lm and VarPow approaches; FspAI is one among the enzymes allowing 100% identification with the VarPow approach.

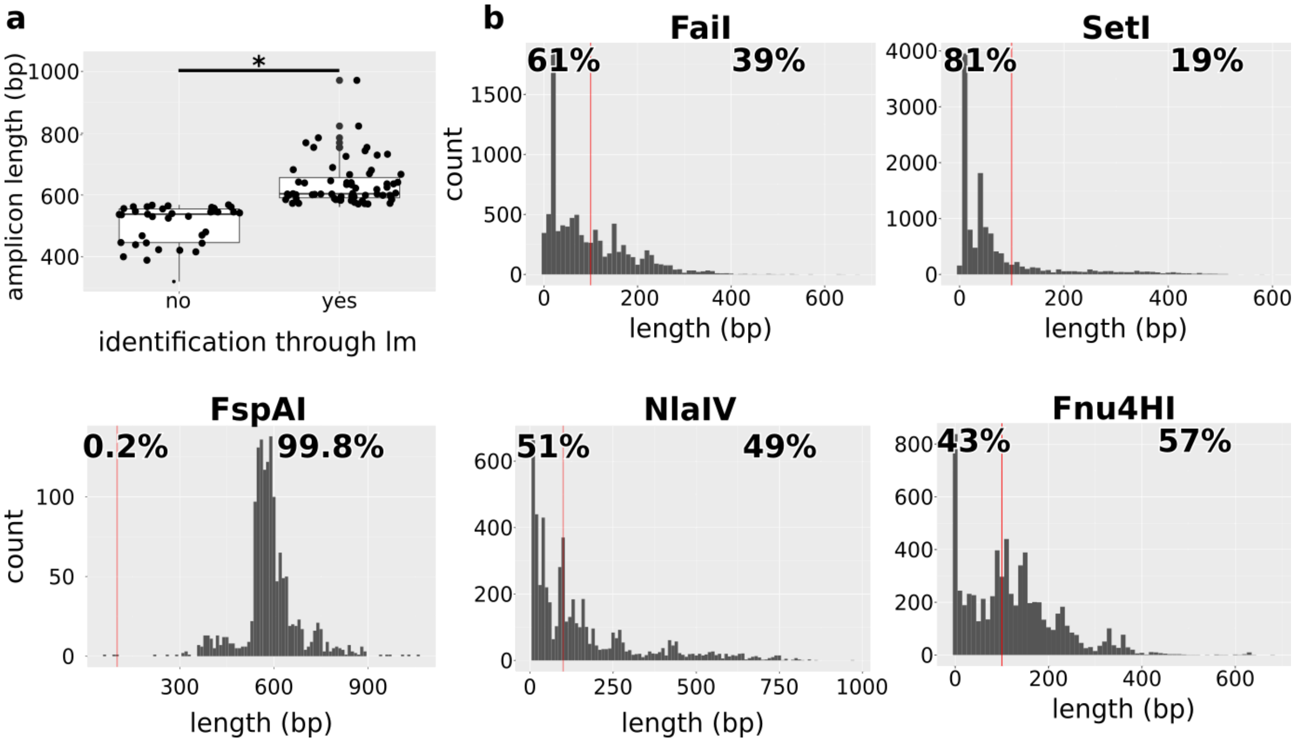

Supplementary figure S7:
Performance of yeast species identification based on two enzyme profiles. a) heatmaps of the percentage of successful identification. For each combination of endonucleases (indicated in the x and y axes), the color indicates the percentage of yeast species identified thanks to the approach indicated above each heatmap; the darker the color the better performance. **b)** heatmaps of the median number of species identified in every query (i.e. the number of False Positive species + the correct species). For each combination of endonucleases (indicated in the x and y axes), the color indicates the percentage of yeast species identified thanks to the approach indicated above each heatmap; the lighter the color the better performance. **c)** heatmaps of precise identifications (i.e. successful identification resulting in the selection of a single species). For each combination of endonucleases (indicated in the x and y axes), the color indicates the percentage of yeast species identified thanks to the approach indicated above each heatmap; the darker the color the better performance.

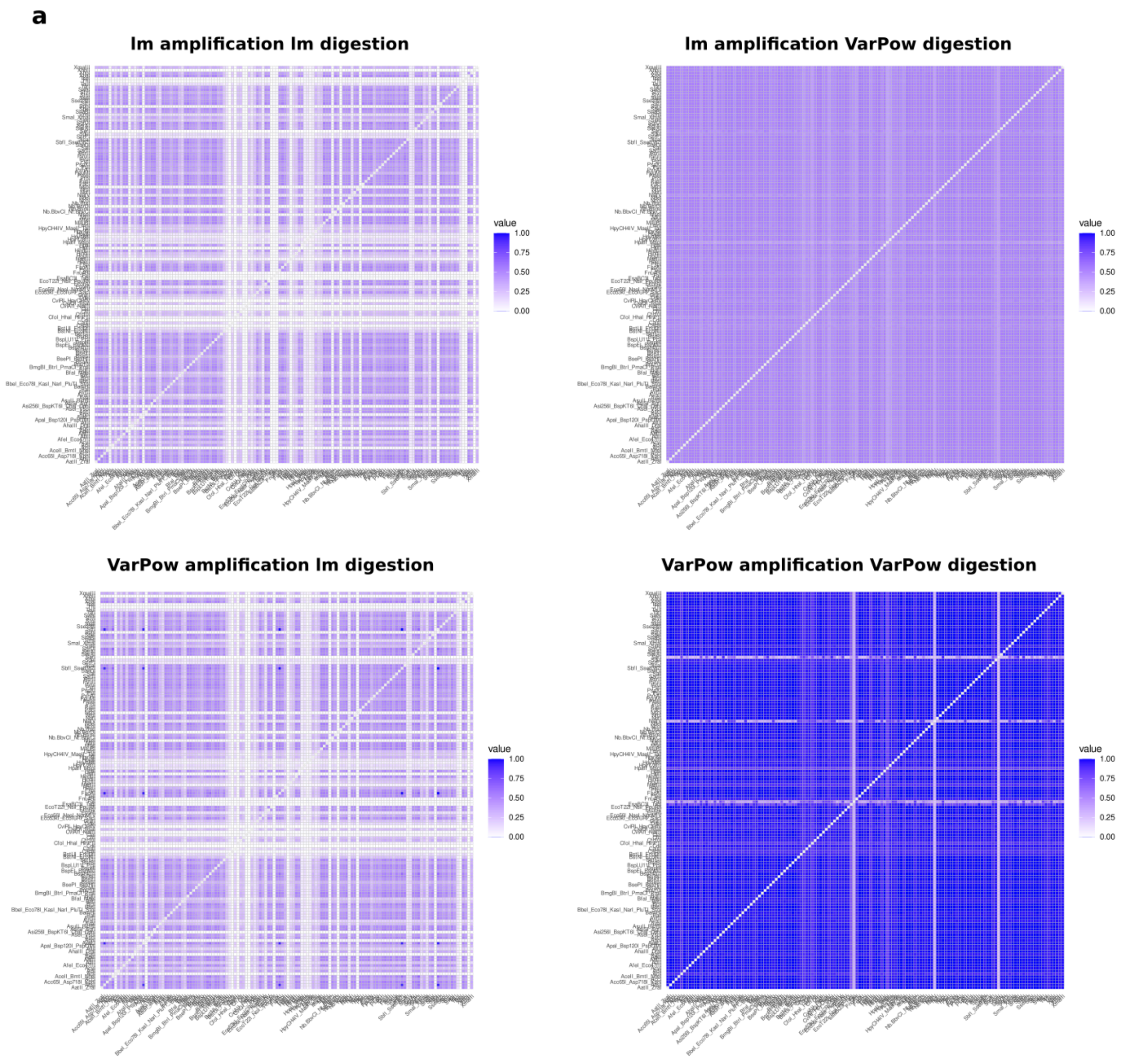
a
lm amplification lm digestion
lm amplification VarPow digestion
VarPow amplification lm digestion
VarPow amplification VarPow digestion
value
1.00
0.75
0.50
0.25
0.00

b

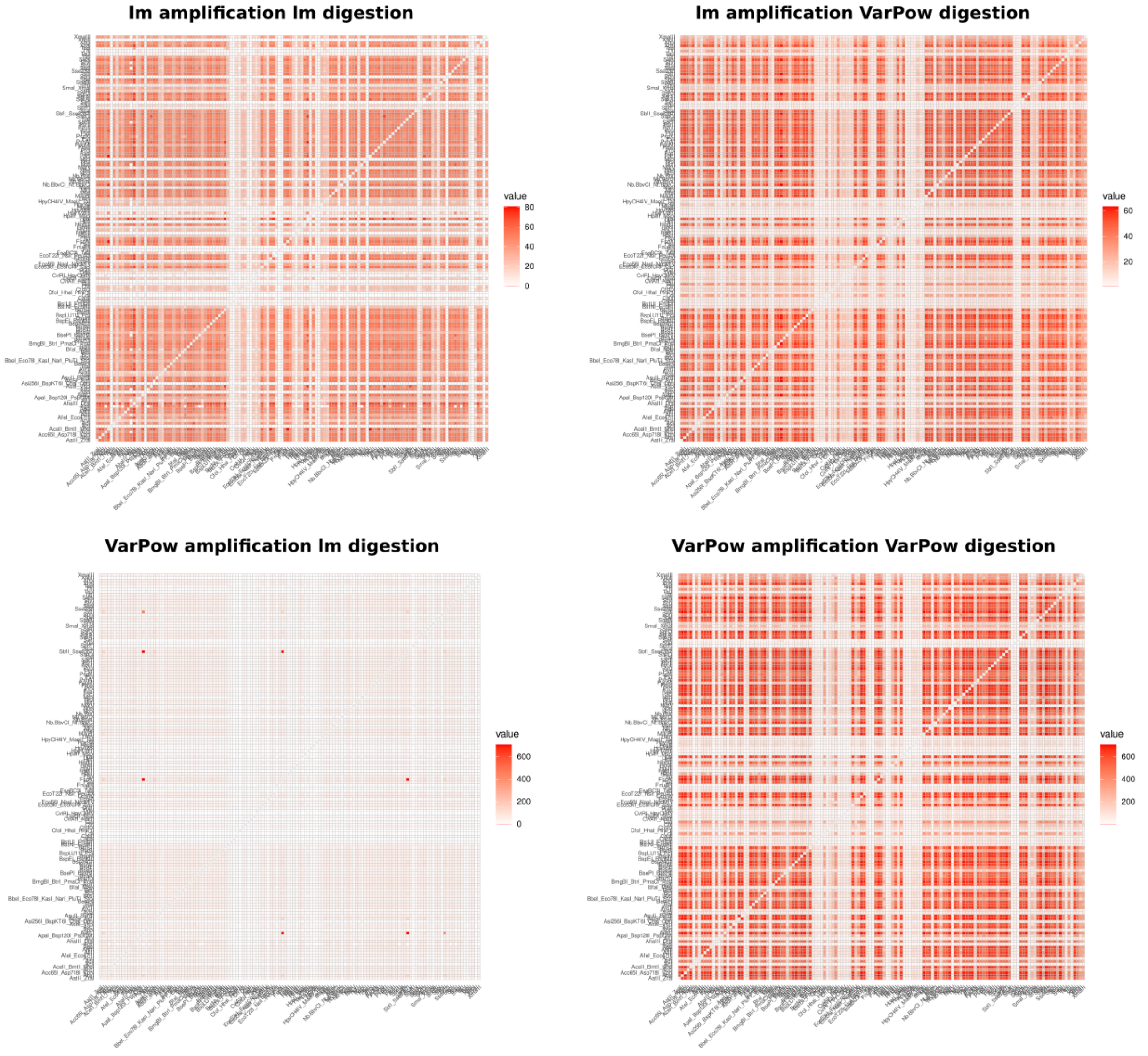
Im amplification Im digestion
value
80
60
40
20
0
Im amplification VarPow digestion
value
60
40
20
VarPow amplification Im digestion
value
600
400
200
0
VarPow amplification VarPow digestion
value
600
400
200

c

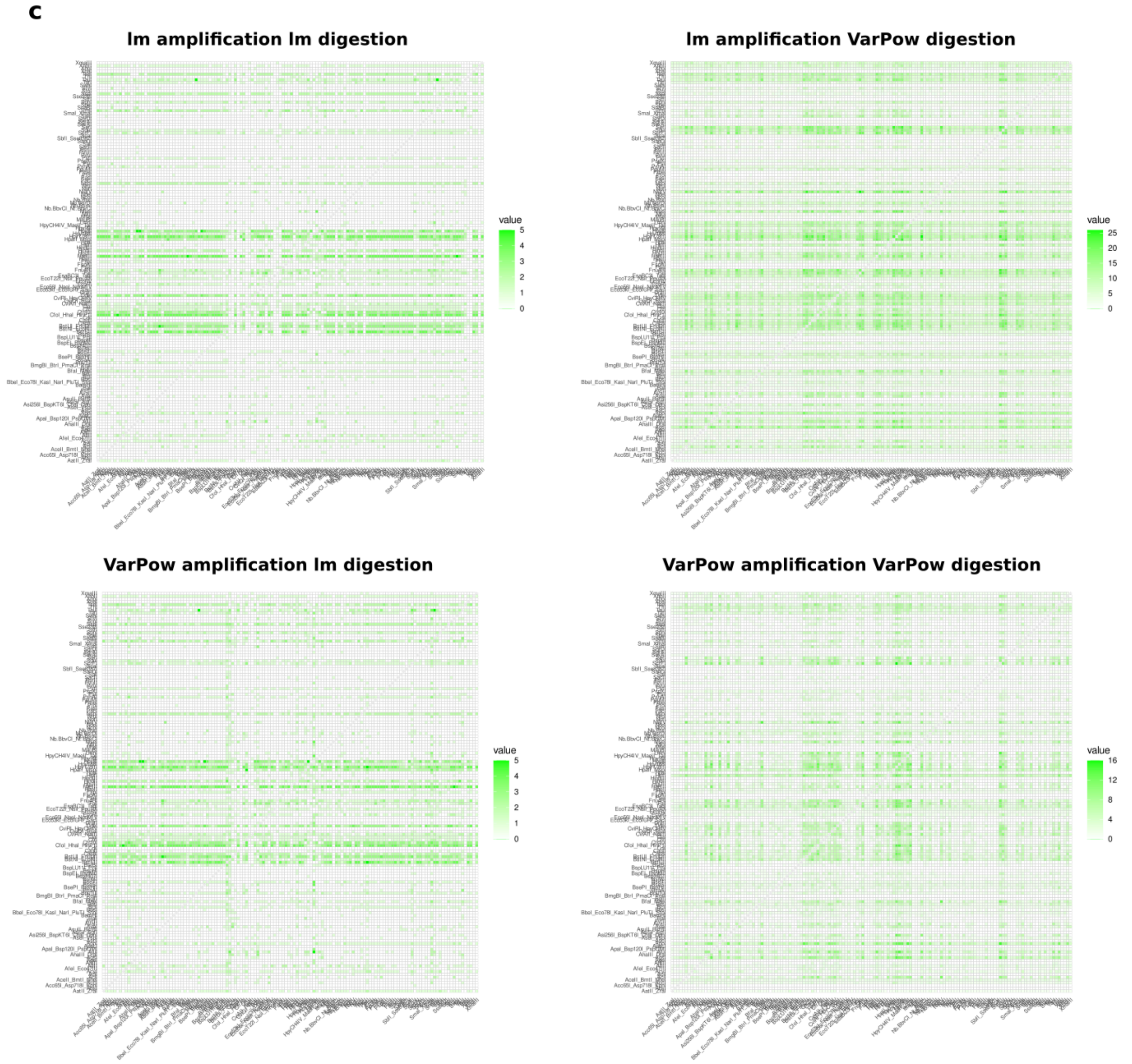
lm amplification lm digestion
lm amplification VarPow digestion
VarPow amplification lm digestion
VarPow amplification VarPow digestion
value

Supplementary information

Supplementary Figure S8: WebLogo [48] representation of the intra-specific conservation of the ITS1-5.8S-ITS2 genomic region sequence. **a)** Sequence logo of 500 *Candida albicans* ITS1-5.8S-ITS2 strains; only the regions presenting sequence variations are shown. **b)** Sequence logo of 500 *Saccharomyces cerevisiae* ITS1-5.8S-ITS2 strains. Black horizontal lines on top of the sequence show the presence of an endonuclease restriction site, indicated by the corresponding enzyme name. Images were generated with WebLogo [44] and then implemented with information on endonuclease restriction sites.

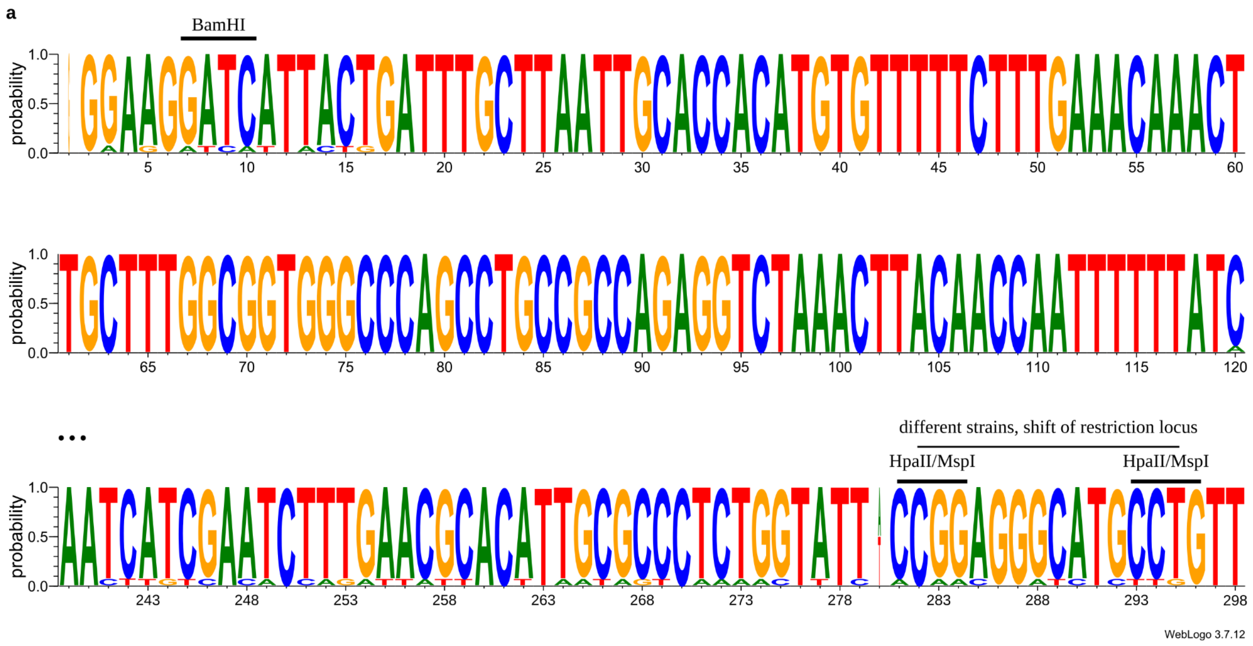

Supplementary information

b

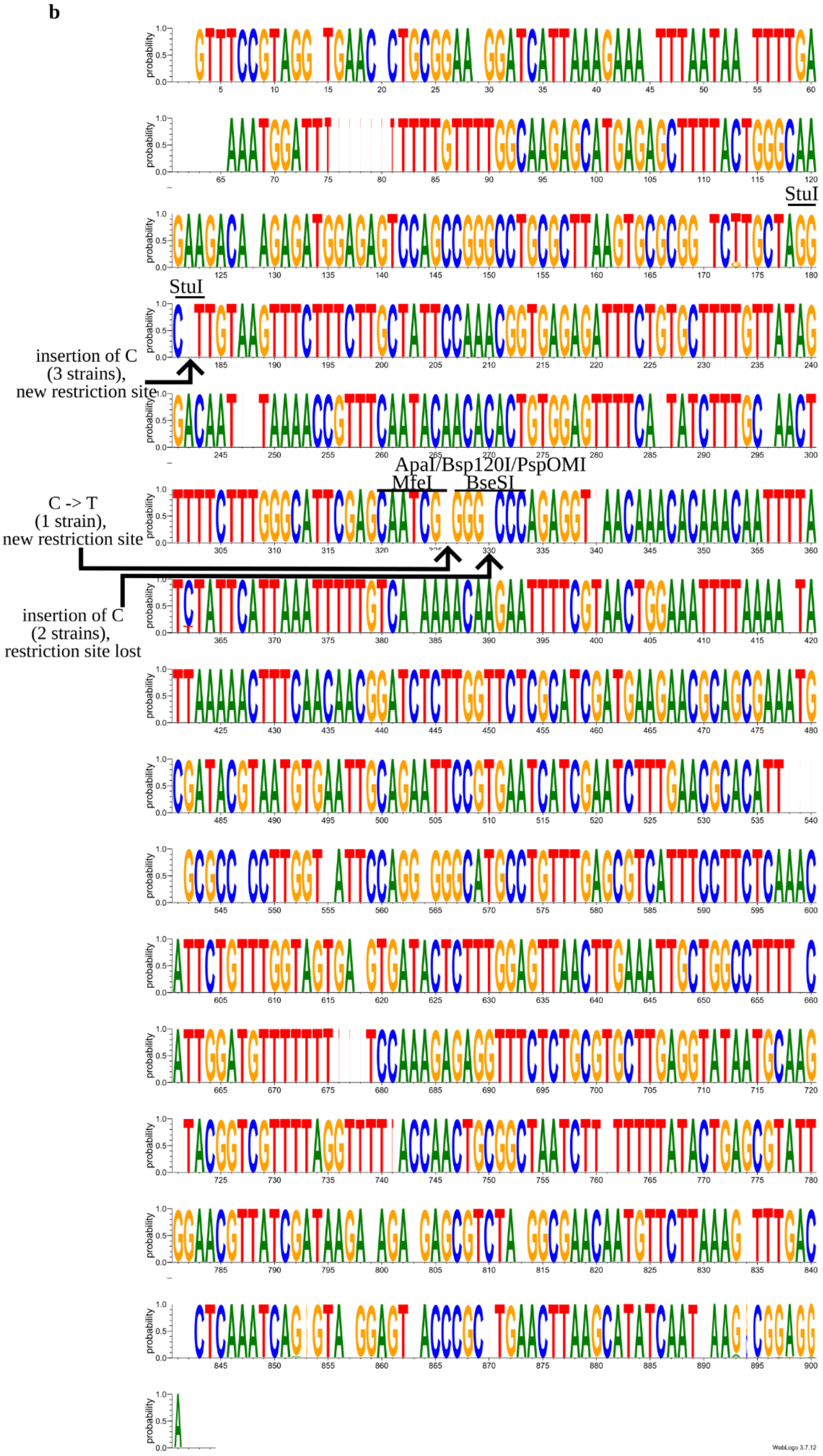

1 Scripts

definition_of_amplicon_size.py

```
##usage python definition_of_amplicon_size.py <input multifasta> <outfile>

import sys
inputs=sys.argv

InFile=inputs[1]
inputFile=open(InFile)
outFileName=inputs[2]
outFile=open(outFileName,"w")

print("preview of the results: ")
for sequences in inputFile:
    if sequences.startswith(">"):
        name=sequences .strip()[1:]
    else:
        sequence=len(sequences)
        name1=name+"\t"+str(sequence)
        outFile.write(name1+"\n")
```

back_to_index

Supplementary information

get_length_of_fragments.py

```
##usage python get_length_of_fragments.py <input multifasta> <outfile> <enzyme>

import sys
import re

inputs=sys.argv

InFile=inputs[1]
inputFile=open(InFile)
restrictionEnzyme=inputs[3]
outFileNameBegin=inputs[2]
outFileName=outFileNameBegin+"_"+restrictionEnzyme+".txt"
outFile=open(outFileName,"w")

EnzymesList={"HaeIII":"GGCC","HinfI":"GA(A|T|C|G)TC","CfoI_HhaI_HinP1I":"GCGC",
"AatII_ZraI":"GACGTC", "AbsI":"CCTCGAGG", "Acc65I_Asp718I_KpnI":"GGTACC",
"AccI":"GT(A|C)(T|G)AC", "AceII_BmtI_NheI":"GCTAGC", "AciI":"(CCGC|GCGG)", "AclI":"AACGTT",
"AcyI":"G(A|G)CG(C|T)C", "AfeI_Eco47III":"AGCGCT", "AflII":"CTTAAG", "AflIII":"AC(A|G)(C|T)GT",
"AgeI":"ACCGGT", "AgsI":"TT(G|C)AA", "AhaIII_DraI":"TTTAAA","AluI":"AGCT",
"ApaI_Bsp120I_PspOMI":"GGGCCC", "ApaLI":"GTGCAC", "ApoI":"(A|G)AATT(C|T)", "AscI":"GGCGCGCC",
"AseI_VspI":"ATTAAT","Asi256I_BspKT6I_ChaI_DpnI":"GATC", "AsiSI_SgfI":"GCGATCGC",
"AsuII_BstBI":"TTCGAA", "AvaI":"C(C|T)CG(A|G)G", "AvaII":"GG(A|T)CC","AvrII":"CCTAGG",
"BalI":"TGGCCA", "BamHI":"GGATCC", "BbeI_Eco78I_KasI_NarI_PluTI_SfoI":"GGCGCC",
"BbsI":"GAAGAC", "BclI":"TGATCA", "BetI":"(A|T)CCGG(A|T)","BfaI_MaeI":"CTAG", "BglII":"AGATCT",
"BmgBI_BtrI_PmaCI_PmlI":"CACGTG", "Bpu10I":"(CCT(A|T|C|G)AGC)|(GCT(A|T|C|G)AGG)",
"BsaAI":"(C|T)ACGT(A|G)", "BsePI_BssHII":"GCGCGC","BseSI":"G(T|G)GC(A|C)C",
"BseYI":"(CCCAGC)|(GCTGGG)", "BssSI-v2_Nb.BssSI":"(CACGAG)|(CTCGTG)", "BsiWI":"CGTACG",
"Bsp1407I":"TGTACA", "BspEI_BspMII":"TCCGGA","BspHI":"TCATGA", "BspLU11I_PciI":"ACATGT",
"BsrBI":"(CCGCTC)|(GAGCGG)","BstEII":"GGT(A|T|C|G)ACC", "BstNI_EcoRII":"CC(A|T)GG",
"BstUI_FnuDII":"CGCG", "Cac8I":"GC(A|T|C|G)(A|T|C|G)GC",
"CauII":"CC(G|C)GG","CdiI":"(CATCG)|(CGATG)", "Cfr10I":"(A|G)CCGG(C|T)", "CfrI":"(C|T)GGCC(A|G)",
"ClaI":"ATCGAT", "CviAII_NlaIII":"CATG", "CviQI_RsaI":"GTAC", "CviRI_HpyCH4V":"TGCA",
"DdeI":"CT(A|T|C|G)AG","DraII":"(A|G)GG(A|T|C|G)CC(C|T)", "Eco53kI_EcoICRI_SacI":"GAGCTC",
"Eco56I_NaeI_NgoMIV":"GCCGGC", "EcoRI":"GAATTC", "EcoRV":"GATATC",
"EcoT22I_NsiI_Ppu10I":"ATGCAT", "EsaBC3I_TaqI":"TCGA", "FaiI":"(C|T)AT(A|G)",
"Fnu4HI":"GC(A|T|C|G)GC","FseI":"GGCCGGCC", "FspAI":"(A|G)TGCGCA(C|T)", "FspI":"TGCGCA",
"HaeII":"(A|G)GCGC(C|T)", "HgiJII":"G(A|G)GC(C|T)C", "HindII":"GT(C|T)(A|G)AC",
"HindIII":"AAGCTT","HpaI":"GTTAAC", "HpaII_MspI":"CCGG", "Hpy178III":"TC(A|T|C|G)(A|T|C|G)GA",
"Hpy188I":"TC(A|T|C|G)GA", "Hpy8I":"GT(A|T|C|G)(A|T|C|G)AC", "Hpy99I":"CG(A|T)CG",
"HpyCH4IV_MaeII_TaiI":"ACGT",
"I-CeuI":"(TAACTATAACGGTCCTAAGGTAGCGAA)|(TTCGCTACCTTAGGACCGTTATAGTTA)",
"I-SceI":"(TAGGGATAACAGGGTAAT)|(ATTACCCTGTTATCCCTA)",
"LmnI":"(GCTCC(A|T|C|G))|((A|T|C|G)GGAGC)",
 "MauBI":"CGCGCGCG", "MfeI":"CAATTG", "MluI":"ACGCGT", "MseI":"TTAA",
"Nb.BbvCI_Nt.BbvCI":"(CCTCAGC)|(GCTGAGG)", "Nb.BsmI":"(GAATGC(A|T|C|G))|((A|T|C|G)GCATTC)",
"Nb.BsrDI":"(GCAATG(A|T|C|G)(A|T|C|G))|((A|T|C|G)(A|T|C|G)CATTGC)",
"Nb.BtsI":"(GCAGTG(A|T|C|G)(A|T|C|G))|((A|T|C|G)(A|T|C|G)CACTGC)", "NcoI":"CCATGG",
"NdeI":"CATATG","NlaIV":"GG(A|T|C|G)(A|T|C|G)CC", "NotI":"GCGGCCGC", "NruI":"TCGCGA",
"NspI":"(A|G)CATG(C|T)",
"PacI":"TTAATTAA", "PasI":"CCC(A|T)GGG", "PfoI":"TCC(A|T|C|G)GGA",
"PI-PspI":"(TGGCAAACAGCTATTATGGGTATTATGGGT)|(ACCCATAATACCCATAATAGCTGTTTGCCA)",
"PI-SceI":"(ATCTATGTCGGGTGCGGAGAAAGAGGTAAT)|(ATTACCTCTTTCTCCGCACCCGACATAGAT)",
```

```
"PmeI":"GTTTAAAC", "PpuMI":"(A|G)GG(A|T)CC(C|T)",
"PshAI":"GAC(A|T|C|G)(A|T|C|G)(A|T|C|G)(A|T|C|G)GTC",
"PsiI":"TTATAA","PspXI":"(A|C|G)CTCGAG(T|C|G)", "PstI":"CTGCAG", "PvuI":"CGATCG",
"PvuII":"CAGCTG", "RsrII":"CGG(A|T)CCG", "SacII":"CCGCGG",
"SalI":"GTCGAC","SanDI":"GGG(A|T)CCC", "SbfI_Sse8387I":"CCTGCAGG", "ScaI":"AGTACT",
"ScrFI":"CC(A|T|C|G)GG", "SduI":"G(A|T|G)GC(A|T|C)C", "SetI":"A(G|C)(G|C)T",
"SexAI":"ACC(A|T)GGT","SgrAI":"C(A|G)CCGG(C|T)G", "SgrDI":"CGTCGACG",
"SimI":"(GGGTC)|(GACCC)", "SmaI_XmaI":"CCCGGG", "SmlI":"CT(C|T)(A|G)AG", "SnaBI":"TACGTA",
"SpeI":"ACTAGT", "SphI":"GCATGC", "SrfI":"GCCCGGGC", "Sse232I":"CGCCGGCG","SspI":"AATATT",
"StuI":"AGGCCT", "StyI":"CC(A|T)(A|T)GG", "SwaI":"ATTTAAAT", "TatI":"(A|T)GTAC(A|T)",
"TauI":"GC(G|C)GC", "TfiI":"GA(A|T)TC","TseI":"GC(A|T)GC",  "XbaI":"TCTAGA", "XhoI":"CTCGAG",
"XhoII":"(A|G)GATC(C|T)", "XmaIII":"CGGCCG"}

CutEnzymes={"HaeIII":"2","HinfI":"1","CfoI_HhaI_HinP1I":"3","AatII_ZraI":"5", "AbsI":"2",
"Acc65I_Asp718I_KpnI":"1", "AccI":"2", "AceII_BmtI_NheI":"5", "AciI":"1", "AclI":"2", "AcyI":"2",
"AfeI_Eco47III":"3", "AflII":"1", "AflIII":"1", "AgeI":"1", "AgsI":"3", "AhaIII_DraI":"3", "AluI":"2",
"ApaI_Bsp120I_PspOMI":"5", "ApaLI":"1", "ApoI":"1", "AscI":"2", "AseI_VspI":"2",
"Asi256I_BspKT6I_ChaI_DpnI":"1", "AsiSI_SgfI":"5", "AsuII_BstBI":"2", "AvaI":"1", "AvaII":"1", "AvrII":"1",
"BalI":"3", "BamHI":"1", "BbeI_Eco78I_KasI_NarI_PluTI_SfoI":"5","BbsI":"6", "BclI":"1", "BetI":"1",
"BfaI_MaeI":"1", "BglII":"1", "BmgBI_BtrI_PmaCI_PmlI":"3", "Bpu10I":"2", "BsaAI":"2", "BsePI_BssHII":"1",
"BseSI":"5", "BseYI":"1", "BssSI-v2_Nb.BssSI":"1", "BsiWI":"1","Bsp1407I":"1", "BspEI_BspMII":"1",
"BspHI":"1", "BspLU11I_PciI":"1","BsrBI":"3","BstEII":"1","BstNI_EcoRII":"2","BstUI_FnuDII":"2", "Cac8I":"3",
"CauII":"1", "CdiI":"4", "Cfr10I":"1", "CfrI":"3", "ClaI":"2", "CviAII_NlaIII":"1", "CviQI_RsaI":"1",
"CviRI_HpyCH4V":"2", "DdeI":"1", "DraII":"2", "Eco53kI_EcoICRI_SacI":"3", "Eco56I_NaeI_NgoMIV":"1",
"EcoRI":"1", "EcoRV":"3", "EcoT22I_NsiI_Ppu10I":"5", "EsaBC3I_TaqI":"2", "FaiI":"2", "Fnu4HI":"2","FseI":"6",
"FspAI":"4", "FspI":"3", "HaeII":"5", "HgiJII":"5", "HindII":"3", "HindIII":"1", "HpaI":"3",
"HpaII_MspI":"1","Hpy178III":"2", "Hpy188I":"3", "Hpy8I":"3", "Hpy99I":"5", "HpyCH4IV_MaeII_TaiI":"1",
"I-CeuI":"17", "I-SceI":"9", "LmnI":"5","MauBI":"2", "MfeI":"1", "MluI":"1", "MseI":"1", "Nb.BbvCI_Nt.BbvCI":"5",
"Nb.BsmI":"5", "Nb.BsrDI":"6", "Nb.BtsI":"6", "NcoI":"1", "NdeI":"2", "NlaIV":"3", "NotI":"2", "NruI":"3",
"NspI":"5", "PacI":"5", "PasI":"2", "PfoI":"1", "PI-PspI":"17", "PI-SceI":"15", "PmeI":"4", "PpuMI":"2",
"PshAI":"6", "PsiI":"3","PspXI":"2", "PstI":"5", "PvuI":"4", "PvuII":"3", "RsrII":"2", "SacII":"4", "SalI":"1",
"SanDI":"2", "SbfI_Sse8387I":"6", "ScaI":"3", "ScrFI":"2", "SduI":"5","SetI":"4", "SexAI":"1", "SgrAI":"2",
"SgrDI":"2", "SimI":"2", "SmaI_XmaI":"3", "SmlI":"1", "SnaBI":"3", "SpeI":"1", "SphI":"5", "SrfI":"4",
"Sse232I":"2","SspI":"3", "StuI":"3", "StyI":"1", "SwaI":"4", "TatI":"1", "TauI":"4", "TfiI":"1", "TseI":"1", "XbaI":"1",
"XhoI":"1", "XhoII":"1","XmaIII":"1"}


sequenceTarget=EnzymesList[restrictionEnzyme]
print(sequenceTarget)
lengthBlunt=CutEnzymes[restrictionEnzyme]
outLine=""
print("preview of the results: ")
for sequences in inputFile:
    if sequences.startswith(">"):
        name=sequences.strip()
    else:
        sequence=sequences.upper()
        if not restrictionEnzyme in ["HinfI", "AccI", "AciI", "AcyI", "AflIII", "AgsI", "ApoI", "AvaI", "AvaII", "BetI",
"Bpu10I", "BsaAI", "BseSI", "BseYI", "BssSI-v2_Nb.BssSI", "BsrBI", "BstEII", "BstNI_EcoRII", "Cac8I",
"CauII", "CdiI", "Cfr10I", "CfrI", "DdeI", "DraII", "FaiI", "Fnu4HI", "FspAI", "HaeII", "HgiJII", "HindII",
"Hpy178III","Hpy188I", "Hpy8I", "Hpy99I", "I-CeuI", "I-SceI", "LmnI",  "Nb.BbvCI_Nt.BbvCI", "Nb.BsmI",
"Nb.BsrDI", "Nb.BtsI", "NlaIV", "NspI", "PasI", "PfoI", "PI-PspI", "PI-SceI", "PpuMI", "PshAI", "PspXI", "RsrII",
"SanDI", "ScrFI", "SduI", "SetI", "SexAI", "SgrAI", "SimI", "SmlI", "StyI", "TatI", "TauI", "TfiI", "TseI", "XhoII"]:
            fragment=sequence.find(sequenceTarget)
            1stFragment=sequence[:fragment+int(lengthBlunt)]
```

```
        remainance=sequence[fragment+int(lengthBlunt):]
        if not fragment==-1:
            outLine=name[1:]+"\t"+str(fragment+int(lengthBlunt))
        else:
            outLine=name[1:]+"\t"+str(len(sequence)-1)
        while not fragment==-1:
            second=remainance.find(sequenceTarget)
            remainance=remainance[second+int(lengthBlunt):]
            if not second==-1:
                outLine=outLine+"\t"+str(second+int(lengthBlunt))
            else:
                outLine=outLine+"\t"+str(len(remainance)-1)
            fragment=second
    else:
        if re.search(sequenceTarget,sequence):
            fragment1=re.search(sequenceTarget,sequence)
            fragment=sequence.find(fragment1.group(0))
            1stFragment=sequence[:fragment+int(lengthBlunt)]
            remainance=sequence[fragment+int(lengthBlunt):]
            outLine=name[1:]+"\t"+str(fragment+int(lengthBlunt))
            while re.search(sequenceTarget,remainance):
                second1=re.search(sequenceTarget,remainance)
                second=remainance.find(second1.group(0))
                remainance=remainance[second+int(lengthBlunt):]
                outLine=outLine+"\t"+str(second+int(lengthBlunt))
            outLine=outLine+"\t"+str(len(remainance)-1)
        else:
            outLine=name[1:]+"\t"+str(len(sequence)-1)
    outFile.write(outLine+"\n")
```

back_to_index

lmErrFunction.R

```
lmErrFunction=function(input_number){
  inputExpUpp=round(((as.numeric(m.0$coefficients[2])*input_number+as.numeric(m.0$coefficients[1]))+2*
        m.0$sigma*sqrt(((1307*(input_number)^2)-(2*A*input_number+B)/denom),2)
  inputExpLow=round(((as.numeric(m.0$coefficients[2])*input_number+as.numeric(m.0$coefficients[1]))-2*m
        .0$sigma*sqrt(((1307*(input_number)^2)-(2*A*input_number+B)/denom),2)
  output=list(inputExpFit,inputExpLow,inputExpUpp)
  names(output)=c("fit","lower","upper")
  return(output)
}
```

back to index

powerErrFunction.R

```
powerErrFunction=function(input_number){
  inputExpFit=round(as.numeric(m.0w$coefficients[2])*input_number+as.numeric(m.0w$coefficients[1]),2)
  inputExpUpp=round((as.numeric(m.0w$coefficients[2])*input_number+as.numeric(m.0w$coefficients[1])+2
        *((as.numeric(powerRes$coefficients[2])*input_number)+as.numeric(powerRes$coefficients[1])),2)
  inputExpLow=round((as.numeric(m.0w$coefficients[2])*input_number+as.numeric(m.0w$coefficients[1]))+2
        *((as.numeric(powerRes$coefficients[2])*input_number)+as.numeric(powerRes$coefficients[1])),2)
   output=list(inputExpFit,inputExpLow,inputExpUpp)
   names(output)=c("fit","lower","upper")
   return(output)
}
```

back to index

select_on_amplicon_length.R

```
select_on_amplicon_length=function(reference,inputExp,model=c("lm","VarPow")){
 if (missing(model)|!model%in%c("lm","VarPow")) model="VarPow"
 referenceTable=read.table(reference, header=F,sep="\t")
 colnames(referenceTable)=c("species","amplicon_size")
 if(model=="lm"){
   temp0=lmErrFunctions(as.numeric(inputExp))
 }else if (model=="VarPow"){
   temp0=powerErrFunctions(as.numeric(inputExp))
 }
 inputExpFit=temp0$fit
 inputExpUpp=temp0$upper
 inputExpLow=temp0$lower
 referenceSel=referenceTable[as.numeric(referenceTable$amplicon_size)>=as.numeric(inputExpLow)&
                    as.numeric(referenceTable$amplicon_size)<=as.numeric(inputExpUpp),]
 nmatches=nrow(referenceSel)
 output=list(referenceSel,nmatches)
 names(output)=c("selected_profiles","n_of_matches")
 return(output)
}
```

back_to_index

select_on_digestion.R

```
select_on_digestion=function(input,referenceSel,listSelAmpl,model=c("lm","VarPow")){
  # input= the profile of digestion, written as i.e. "536+218+150"
  # referenceSel = the path to the file with the results of enzyme digestion
  # listSelAmpl=listSelAmpl$selected_profiles$species, output of select_on_amplicon_length.R
  if (missing(model)|!model%in%c("lm","VarPow")) model="VarPow"
  referenceEnzTable=read.table(referenceSel, header=F,sep="\t",row.names=1)
  EnzChosen=referenceEnzTable[row.names(referenceEnzTable)%in%listSelAmpl$species,]
  if(is.data.frame(EnzChosen)){
    EnzChosen1=t(apply(EnzChosen,1,function(x) sort(x,decreasing = T)))
    EnzChosen1[EnzChosen1<100]=0
    EnzChosen1=EnzChosen1[,colSums(EnzChosen1)!=0]
  }else{EnzChosen1=EnzChosen}
  inputVals=unlist(strsplit(input,split="[+]"))
  if(is.matrix(EnzChosen1)){
    summaryResults=matrix(0,nrow=nrow(EnzChosen1),ncol=2)
    row.names(summaryResults)=row.names(EnzChosen1)
    colnames(summaryResults)=c("matching_fragments","expected_fragments")
    summaryResults[,2]=apply(EnzChosen1,1,function(x) length(x[x!=0]))
  }else{
    summaryResults=matrix(0,nrow=length(EnzChosen1),ncol=2)
    row.names(summaryResults)=names(EnzChosen1)
    colnames(summaryResults)=c("matching_fragments","expected_fragments")
    summaryResults[,2]=length(EnzChosen1[EnzChosen1!=0])
  }
  for(fragments in 1:length(inputVals)){
    if(model=="lm"){
      temp0=lmErrFunctions(as.numeric(inputVals[fragments]))
    }else if (model=="VarPow"){
      temp0=powerErrFunctions(as.numeric(inputVals[fragments]))
    }
    inputExpFit=temp0$fit
    inputExpUpp=temp0$upper
    inputExpLow=temp0$lower
    if(inputExpLow>100){
      if(is.matrix(EnzChosen1)){
        tempSum=t(apply(EnzChosen1,1,function(x) ifelse(x>=inputExpLow&x<=inputExpUpp,+1,+0)))
summaryResults[,1]=summaryResults[,1]+as.numeric(ifelse(rowSums(tempSum)>=1,1,0))
      }else{
        tempSum=ifelse(EnzChosen1>=inputExpLow&EnzChosen1<=inputExpUpp,+1,+0)
summaryResults[,1]=summaryResults[,1]+as.numeric(ifelse(sum(tempSum)>=1,1,0))
      }
    }
  }
  summaryResults=as.data.frame(summaryResults)
  summaryResults$perc=summaryResults[,1]/summaryResults[,2]
  summaryResults1=summaryResults[summaryResults$perc<=1&summaryResults$perc!=0,]

summaryResults1=summaryResults1[(summaryResults1$matching_fragments/length(inputVals))==max((su
mmaryResults1$matching_fragments/length(inputVals))),]
  return((summaryResults1))
}
```
